# Celastrol modulates IRS1 expression to alleviate ovarian aging and to enhance follicular development

**DOI:** 10.1007/s10565-025-10079-7

**Published:** 2025-09-02

**Authors:** Yao Jiang, Yonghua Shi, Meng Lv, Tao Wang, Penghao Wang, Xiaolong Yuan, Fei Gao, Bin Ma

**Affiliations:** 1 School of Medical, Molecular and Forensic Sciences, Murdoch University, Murdoch, WA Australia; 2 Centre for Healthy Ageing, Health Futures Institute, Murdoch University, Murdoch, WA Australia; 3 State Key Laboratory of Swine and Poultry Breeding Industry, Guangdong Provincial Key Lab of Agro-Animal Genomics and Molecular Breeding, College of Animal Science, Guangdong Laboratory of Lingnan Modern Agriculture, National Engineering Research Center for Breeding Swine Industry, South China Agricultural University, Guangzhou, Guangdong China; 4 Sun Yat-Sen University Cancer Center, Sun Yat-Sen University, Zhongshan, Guangdong China; 5 The Kids Research Institute Australia, Nedlands, WA Australia; 6 Centre for Crop and Food Innovation, Food Future Institute, Murdoch University, Murdoch, WA Australia; 7 Shenzhen Branch, Guangdong Laboratory for Lingnan Modern Agriculture, Agricultural Genomics Institute at Shenzhen, Genome Analysis Laboratory of the Ministry of Agriculture, Chinese Academy of Agricultural Sciences, Shenzhen, Guangdong China

**Keywords:** Celastrol, IRS1, Ovarian aging, Oocyte maturation, Follicular development, Natural product

## Abstract

Ovarian aging significantly contributes to the decline of the female reproductive system, adversely affecting fertility and endocrine homeostasis. To address the challenges posed by reproductive aging, natural products have shown promising preventive and therapeutic effects. Here, we investigated the beneficial effects of natural compound celastrol on ovarian development and aging, together with its underlying mechanisms. We found that celastrol administration at a concentration of 3 mg/kg promoted follicle development in young mice and enhanced porcine oocyte maturation, while regulating granulosa cell proliferation and apoptosis. In 12-month-old mice (equivalent to middle-aged adults), celastrol exhibited similar beneficial effects. Transcriptomic analysis revealed that differentially expressed genes post-celastrol treatment were associated with steroid biosynthesis, estrogen signaling pathways, type 2 diabetes, insulin secretion, meiosis, and apoptosis. Additionally, insulin receptor substrate 1 (IRS1), an adapter protein in insulin signaling, was shown to advance puberty in young mice and to facilitate oocyte maturation. Overexpression of IRS1 in oocytes promoted follicular development and oocyte maturation, resulting in enhanced steroid hormone levels, whereas IRS1 knockdown inhibited these processes. Our findings indicate that celastrol may regulate ovarian development and aging by modulating IRS1 expression and its related pathways, suggesting celastrol as a novel small-molecule compound targeting IRS1, and offering new perspectives for potential therapeutic strategies against reproductive aging and infertility.

**Supplementary Information:**

The online version contains supplementary material available at 10.1007/s10565-025-10079-7.

## Introduction

Global infertility affects millions of couples worldwide, with around 17% of couples experiencing conception problems (Bagade et al. 2022; Feng et al. 2025). Factors contributing to infertility include maternal age, lifestyle choices, environmental factors, and medical conditions (Bayoumi et al. 2024). Ovarian aging is a biomarker of reproductive system decline in females, characterized by diminished ovarian reserve, decreased oocyte quality, hormonal imbalance, and reduced fertility (Tatone & Amicarelli 2013). Advanced maternal age is linked to the aging of oocytes, which increases the risks of early miscarriage, perinatal mortality, and various congenital disabilities (Mikwar et al. 2020; Moghadam et al. 2022; Ntostis et al. 2021; Zhang et al. 2020). The aging of female oocytes is a critical issue directly related to reproductive health and the occurrence of congenital disabilities, making it a pressing concern for national development and public health (Bayoumi et al. 2024). Therefore, the mechanisms underlying follicular development/oocyte aging need to be elucidated, and strategies to delay this process should be explored.

Ovarian development and oocyte maturation are essential processes of the female reproductive system and are intricately regulated by a complex network of signaling pathways, including the insulin signaling pathway (Dupont & Scaramuzzi 2016; Liang et al. 2025), the mammalian target of rapamycin (mTOR) signaling pathway (Yap et al. 2008), and oxidative stress-related pathways (Yan et al. 2022). Studies have shown that excessive reactive oxygen species (ROS) can induce DNA damage in oocytes, impair mitochondrial function, and trigger ovarian granulosa cells (GCs) apoptosis, thereby affecting follicular development (Chen et al. 2024; Feng et al. 2022).

The insulin signaling pathway primarily activates the insulin receptor substrate 1 (IRS1)/phosphatidylinositol 3-kinase (PI3K)/AKT pathway through the insulin receptor (Horita et al. 2016). This activation influences downstream signaling molecules such as mTOR and Forkhead box class O 3a (FOXO3a), which regulates the proliferation, differentiation, and survival of ovarian GCs (Yap et al. 2008). However, insulin sensitivity declines with aging, leading to local ovarian insulin resistance, which affects follicular development and accelerates ovarian aging (Tian et al. 2025). IRS1 serves as a key mediator of the insulin signaling pathway. Upon activation of the insulin receptor or insulin-like growth factor 1 receptor (IGF1R), IRS1 undergoes phosphorylation, initiating downstream signaling cascades, including the PI3K-AKT and mitogen-activated protein kinase (MAPK) pathways. During follicular development, IRS1 significantly influences mitochondrial function in oocytes, enhancing ATP production and supporting the meiotic process (Puengpan et al. 2024). Dysregulation of IRS1 can disrupt insulin signaling, ultimately impairing ovarian function. For instance, patients with polycystic ovary syndrome often exhibit reduced levels of IRS1 phosphorylation, which contributes to insulin resistance and ovarian dysfunction (Mu et al. 2021).

Numerous studies have explored potential strategies to delay ovarian aging. These strategies include the use of antioxidants such as resveratrol and melatonin, metabolic regulators such as metformin, and small-molecule compounds that target the mTOR/Sirtuin 1 (SIRT1) signaling pathways (Bharath et al. 2020; Jin et al. 2025; Li et al. 2023; Reiter et al. 2023; Zhu et al. 2023). Celastrol is a natural triterpene compound derived from the root of *Tripterygium wilfordii* and has demonstrated both preventive and therapeutic properties for various conditions, including autoimmune diseases (e.g., rheumatoid arthritis, systemic lupus erythematosus, and Crohn’s disease), metabolic disorders (e.g., obesity and diabetes), cardiovascular diseases (e.g., chronic heart failure, atherosclerosis, and cerebrovascular diseases), and cancer (He et al. 2024; Hu et al. 2020; Li et al. 2025; Li et al. 2022; Pinna et al. 2004; Tan et al. 2024; Xie et al. 2024; Zeng et al. 2024; W. Zhang et al. 2024; Zhou et al. 2023). Recent studies have indicated that celastrol enhances mitochondrial function by activating the PI3K-AKT signaling pathway and improves resistance to oxidative stress through the upregulation of SIRT1 expression (Bakar & Tan 2017; Zhang et al. 2017). Furthermore, it has been shown to mitigate high glucose-induced apoptosis, lactate dehydrogenase release, and the generation of ROS (Zhan et al. 2018). In terms of reproductive health, celastrol has been linked to improved fertility in mice by reducing the expression of inflammatory genes in GCs (Song et al. 2021). As a result, celastrol might offer potential benefits for enhancing ovarian reserve function and improving oocyte quality. In this study, we aimed to investigate the effects of celastrol and IRS1 on ovarian development/aging, and their potential association and interaction during these processes.

## Materials and methods

### Animal treatment

Female *C57BL/6J* mice were obtained from the Sun Yat-sen University Cancer Center (Guangzhou, China). All mice were kept in individually ventilated filter cages and had unrestricted access to autoclaved food and water. They were maintained under specific pathogen-free conditions, with a 12-h light/dark cycle, a room temperature of 25 °C, and a relative humidity of 60%. Daily monitoring of the mice was conducted to observe any changes in body weight and to check for signs/symptoms of illness throughout the duration of the experiment. After the treatment period, the mice were euthanized through CO₂ inhalation, followed by decapitation.

For the administration of celastrol (HY-13067, MedChemExpress, Monmouth Junction, NJ, USA), intraperitoneal injection was employed due to its superior controllability compared to oral administration, enabling precise management of dosage and timing. This approach effectively minimized fluctuations in drug efficacy that could result from variations in the animals’food intake (Gaines Das & North 2007).

A total of 40 three-week-old female *C57BL/6J* mice were randomly assigned to five groups (n = 8 per group): a control group and four celastrol treatment groups, each being administered different concentrations of celastrol (0.5, 1, 3, and 6 mg/kg). Additionally, twelve 12-month-old female mice were randomly divided into two groups (n = 6 per group): a control group and a celastrol treatment group (3 mg/kg). The mice received celastrol or an equivalent volume of dimethyl sulfoxide (DMSO; HY-YO320, MedChem Express) via intraperitoneal injection every other day for three consecutive weeks. For subsequent analyses, 3 to 4 mice from each group were randomly selected/euthanized for further analysis.

Moreover, 32 three-week-old female *C57BL/6J* mice were randomly allocated into four groups: the lentiviral vectors encoding *IRS1* overexpression (*LV-IRS1*) group (n = 8), the Lentiviral Vector-Negative Control (*LV-NC*) group (n = 8), the short hairpin RNA -mediated *IRS1* knockdown (*sh-IRS1*) group (n = 8), and the *shRNA*-Negative Control (*sh-NC*) group (n = 8). Lentiviral vectors (Gene Ray Biotech, Guangzhou, China) were administered at a dose of 1 × 10^7 transducing units (TU) every other day via intraperitoneal injection for three consecutive weeks. For subsequent analyses, 3 to 4 mice from each group were randomly selected/euthanized for further analysis.

### Isolation and culture of GCs

Porcine GCs were isolated and cultured following our previously established protocols (Jiang et al. 2020). Fresh ovaries for isolation of GCs and oocytes were obtained from Large White pigs (180-day-old sows in the early stages of estrus) at a certified slaughterhouse in Guangzhou, China. GCs were extracted from antral follicles (AFs) measuring 3–5 mm in diameter and were cultured in Dulbecco’s Modified Eagle Medium (DMEM)/F12 (Hyclone, Logan, UT, USA) enriched with 10% fetal bovine serum (FBS; Hyclone) at 37 °C in a humidified incubator with 5% CO₂. Once the cells reached 70%–80% confluence, they were treated with celastrol at a concentration of 0.5 μM for 24 h. Following the treatment, protein and RNA were extracted for subsequent analysis.

### Microinjection

mRNAs utilized for microinjection were synthesized by Dongze Biotechnology (Guangzhou, China). In vitro synthesized mRNA fragments of Overexpression Insulin Receptor Substrate 1 (*OE-IRS1*), Small Interfering RNA-Negative Control (*Si-NC*), and Small Interfering RNA Insulin Receptor Substrate 1 (*Si-IRS1*) were microinjected into the cytoplasm of porcine oocytes, whereas the Overexpression Negative Controls (*OE-NC*) were microinjected with sterile water. The primers utilized for synthesizing the siRNA were as follows: upstream primer-*GCCUAUGCCAGCAUCAGUUTT*, and downstream primer-*AACUGAUGCUGGCAUAGGCTT*.

Microinjection was performed using an Olympus IX2-ILL100 Inverted Microscope (Olympus, Tokyo, Japan) equipped with a DMP-400 Microinjection Pump System (Micrology Precision Instruments, Wuhan, China). Porcine oocytes were placed in droplets containing 7.5% polyvinylpyrrolidone (PVP; V900010, Sigma, St. Louis, MO, USA). Holding pipettes (inner diameter ~ 15 μm) were prepared using Pasteur pipettes (747720, BRAND, Wertheim, Germany) with a Narishige PN-30 Magnetic Glass Microelectrode Microneedle Horizontal Puller (Narishige, Kyoto, Japan) and were used to immobilize the oocytes. The injection pipette (inner diameter ~ 1 μm) was employed to aspirate diluted mRNA solution and subsequently to penetrate the oocyte cytoplasm in order to deliver approximately 5–10 pL of the solution. After microinjection, oocytes were transferred into M199 culture medium (C11150500BT, Thermo Fisher Scientific, Waltham, MA, USA) and incubated at 37 °C for 2 h to recover. Subsequently, they were subjected to in vitro maturation (as indicated by the extrusion of the first polar body (PB-1)) and further activity assessments.

### Oocyte collection and in vitro maturation

Porcine healthy cumulus-oocyte complexes (COCs) were harvested from AFs measuring 3–5 mm in diameter using a stereomicroscope. The COCs were then rinsed in Dulbecco′s phosphate-buffered saline supplemented with Polyvinylpyrrolidone (DDPS-PVP; V900010, Sigma) to ensure purity. They were subsequently cultured in M199 maturation medium (C11150500BT; Thermo Fisher Scientific) that contained 10 IU/mL pregnant mare serum gonadotropin (PMSG; P9970, Solarbio, Beijing, China), 10 IU/mL human chorionic gonadotropin (HCG; C805163, Macklin, Shanghai, China), 10 ng/mL epidermal growth factor (EGF; HY-P7109, MedChem Express), 0.1 mg/mL L-cysteine (1206GR050, BioFroxx, Guangzhou, China), and porcine follicular fluid obtained from follicles larger than 6 mm in diameter. The culture system was overlaid with mineral oil (BS927, Biosharp, Guangzhou, China) and maintained at 38.5 °C in a 5% CO₂ atmosphere to facilitate optimal maturation conditions. After 48 h of in vitro maturation, cumulus cells were removed using 1 mg/mL hyaluronidase (H8030, Solarbio), allowing the collection of denuded oocytes. A total of 400 porcine oocytes at the metaphase II (MII) stage were then assessed for maturation rates and used for subsequent RNA extraction.

### EdU proliferation assay

Cell proliferation was evaluated using the Cell-Light™ EdU Kit (RiboBio, Guangdong, China) in accordance with the manufacturer’s guidelines. GCs were plated in 48-well plates and treated with celastrol at a concentration of 0.5 μM for 24 h. Following treatment, 100 μL EdU solution (50 μM) was added to each well and incubated for 2 h to allow for incorporation into the proliferating cells. After incubation, the cells were fixed with 4% paraformaldehyde (PFA; Dingguo Biotech, Guangzhou, China) for 30 min at room temperature. Subsequently, the cells were permeabilized using 100 μL 0.5% Triton X-100 (P0096, Beyotime Biotech, Shanghai, China) for 10 min. The incorporated EdU was detected by Apollo staining (RiboBio), whereas the nuclei were counterstained with Hoechst 33342 (RiboBio) for 30 min in the dark to enhance visualization.

Images were captured using a Nikon ECLIPSE Ti2 fluorescence microscope (Nikon, Tokyo, Japan). To quantify cell proliferation, the ratio of EdU-positive cells to the total number of Hoechst-stained nuclei was calculated from three randomly selected fields per well.

### Cell apoptosis assay

An Annexin V-Fluorescein isothiocyanate (FITC)/propidium iodide (PI) apoptosis detection kit (Liankebio, Shanghai, China) was utilized to assess cell apoptosis (Jiang et al. 2020; Yuan et al. 2018). GCs were cultured in six-well plates and allowed to reach approximately 50% confluence. At this point, the culture medium was replaced with either celastrol at a concentration of 0.5 μM or DMSO as a control. After a 24-h treatment period, the cells were harvested and resuspended in 500 μL of 1 × Annexin V binding buffer. To facilitate apoptosis detection, 5 μL Annexin V-FITC and 5 μL PI were added to the cell suspension. The mixture was gently vortexed and incubated in the dark for 15 min. Following incubation, the apoptotic cells were analyzed using a NovoCyte D2060R Flow Cytometer (BD Biosciences, San Jose, CA, USA).

### RNA extraction and quantitative Real-Time PCR

Total RNA was extracted from a variety of samples, including 42-day-old mouse ovaries (comprising 21 days of age plus 21 days of treatment), ovaries from 13-month-old mice (comprising 12 months of age plus 3 weeks of treatment), and GCs/oocytes from 180-day-old Large White pigs. The extraction was performed using TRIzol reagent (Thermo Fisher Scientific) in accordance with the manufacturer's instructions. The isolated RNA was then reverse-transcribed into complementary DNA (cDNA) utilizing the RevertAid First Strand cDNA Synthesis Kit (Yisheng, Shanghai, China). Quantitative real-time PCR (qRT-PCR) was conducted on a Bio-Rad CFX96 Touch Real-Time PCR System (Bio-Rad; Hercules, CA, USA) employing SYBR Green qPCR Master Mix for amplification. Gene expression levels were quantified using the 2^ − ΔΔCt method. The sequences of primers (Generaybio, Shanghai, China) are shown in Table 1.

**Table 1:** Primers for qRT-PCR

Gene	Primer sequence (5' to 3')	Gene	Primer sequence (5' to 3')
*PCNA*	F:GGTTACTGAGGGCGAGAAGC	*IRS1*	F:TGTCACCCAGTGGTAGTTGCTC
	R:GACCGGCTGAGACTTGCGTA		R:CTCTCAACAGGAGGTTTGGCAG
*STAR*	F:GCTCTCTACTCGGTTCTCGG	*SIRT1*	F:ACCACCCACACCTCTTAAT
	R:TTCCACTCCCCCATTGCTTC		R:GACTCTCCATCGGTTCTTT
*p53*	F:AAGTCTAGAGCCACCGTCCA	*SIRT6*	F: TGGACAATGGAGGAGCGAG
	R:CAGTCTGGCTGCCAATCCA		R: GACCAGGAAGCGGAGGAGG
*casp3*	F:CCAAAGATCATACATGGAAGCG	*P16*	F:GCGCCGTCTCTTGATTACTG
	R:CTGAATGTTTCCCTGAGGTTTG		R:CTGGCTCCTCACTAGCAACA
*casp9*	F:GGCTGGTGGAAGAGCTGC	*P21*	F:ACGTCTCAGGAGGACCATGT
	R:GAGCCTGCCCGCTGGA		R:AGAAGATCAGCCGGCGTTTG
*casp8*	F:GTCTGTACCTTTCTGGCGGA	*BCL*	F:CCTGTGGATGACTGAGTACCTG
	R:CACAACTCCTCCCCTTTGCT		R:AGCCAGGAGAAATCAAACAGAGG
*BCL-2*	F: CTTTGAGTTCGGTGGGGTCA	*GDF9*	F:GTCACCTCTACAATACCGTCCG
	R: GGGCCGTACAGTTCCACAAA		R:TAAACAGCAGGTCCACCATCGG
*SOD1*	F: GATTCTGTGATCGCCCTCT	*BMP15*	F:GATTGGAGCGAAAATGGTGAGGC
	R: CAGCATTTCCCGTCTTTGT		R:GCTACCTGGTTTGATGCTAGAGG
*GAPDH*	F:GGTCGGAGTGAACGGATTT		
	R:CCATTTGATGTTGGCGGGA		

### Western blotting

Western blot analysis was conducted following the protocol established in our previous study (Jiang et al. 2020). Ovary samples were snap-frozen with liquid nitrogen. Once completely frozen, the samples were transferred to cryogenic tubes and stored at −80 °C for long-term preservation until needed for analysis. Protein samples were denatured and then subjected to electrophoresis using sodium dodecyl sulfate–polyacrylamide (SDS-PAGE) gels. The amount of protein loaded onto the gels was 15 μg. After electrophoresis, the proteins were transferred to polyvinylidene difluoride (PVDF; Bio-Rad) membranes. The membranes were subsequently blocked with skimmed milk and incubated overnight at 4 °C with the following primary antibodies: steroidogenic acute regulatory protein (STAR; DF6192, Abcam, Waltham, MA, USA; 1:1000), B-cell Lymphoma (BCL; 26593–1-AP, Proteintech, Rosemont, IL, USA; 1:1000), Proliferating cell nuclear antigen (PCNA; 10205–2-AP, Proteintech, 1:20,000), BCL-2-associated X protein (BAX; Proteintech, 1:5000), IRS1 (17509–1-AP, Proteintech, 1:5000), Caspase-3 (66470–2-Ig, Proteintech, 1:1000), Caspase-8 (13423–1-AP, Proteintech, 1:1000), Caspase-9 (10380–1-AP, Proteintech, 1:1000), P16 (10883–1-AP, Proteintech, 1:2000), SIRT1 (13161–1-AP, Proteintech, 1:3000), Sirtuin 6 (SIRT6; 13572–1-AP, Proteintech, 1:2000), Superoxide Dismutase 1 (SOD1; ab13498, Abcam, 1:5000), and α-Tubulin (11224–1-AP, Proteintech, 1:8000). The membranes were incubated with horseradish peroxidase (HRP)-conjugated goat anti-rabbit IgG secondary antibody (#3032, SAB, Nanjing, China; 1:10,000) for 1 h at room temperature. Following incubation, luminol (Thermo Fisher Scientific) was applied as the chemiluminescent substrate. The images were captured using a ChemiScope 6200 Chemiluminescence Imaging System (Clinx Science Instruments, Shanghai, China). Band intensities were quantified with the ImageJ program (V1.8.0.112; https://imagej.net/ij/).

### Enzyme-linked immunosorbent assay

Enzyme-linked immunosorbent assay (ELISA) was conducted to quantify serum levels of estradiol (E2), follicle-stimulating hormone (FSH), and luteinizing hormone (LH) in mice, utilizing commercially available ELISA kits from Enzyme-linked Biotech (Shanghai, China). Blood was collected from mice using the retro-orbital bleeding method and promptly placed into a collection tube without anticoagulants. After permitting the blood to sit at room temperature for 20 to 30 min, it was centrifuged at 3,000 rpm for 10 min. The supernatant was then carefully transferred to a new tube for subsequent analysis.

The assay began with the addition of an HRP-labeled antibody in the wells of microplates, which formed a complex with the corresponding hormones in the serum samples, and standard solutions of various concentrations. The experimental serum samples (10 μl) and standards were added to designated wells of pre-coated microplates and incubated at 37 °C for 30 min. Following this incubation, the wells were washed to remove unbound components. Then, 100 μL of the antibody-antigen-enzyme complex solution was added to each well and incubated at 37 °C for an additional 30 min. After another washing step, 50 μL of substrate solutions A and B were added sequentially to each well, followed by a 10-min incubation at 37 °C in the dark to allow color development. The reaction was terminated by adding 50 μL of the stop solution, and the optical density (OD) was measured at 450 nm by using an iMark microplate reader (Bio-Rad).

### Hematoxylin and eosin staining and TUNEL assay

Hematoxylin and eosin (H&E) staining was conducted on paraffin sections of ovaries obtained from mice aged 42 days (3 weeks old plus 21 days of treatment) and 13 months (12 months old plus 3 weeks of treatment). The tissue processing and sectioning procedures were conducted as previously described in our study (Quan et al. 2024). Serial paraffin sections (3 μm thick) were prepared using a Leica RM2016 Microtome (Leica Microsystems, Shanghai, China). H&E staining was performed as previously described (Quan et al. 2024). The sections were then examined using a NIKON ECLIPSE E100 microscope (Nikon), and images were captured/analyzed using a NIKON DS-U3 imaging system (Nikon).

The terminal deoxynucleotidyl transferase dUTP Nick End Labeling (TUNEL) assay was conducted with a TUNEL apoptosis detection kit (Servicebio, Wuhan, China). After deparaffinization, the tissue sections were treated with proteinase K for 10 min, followed by three washes with phosphate-buffered saline (PBS; G0002, Servicebio), each lasting 5 min. Once the sections had slightly dried, a membrane-permeabilizing solution (Triton X-100; G1204, Servicebio) was applied, and the slides were incubated at room temperature for 20 min. After a brief drying period, a buffer was added to cover the tissue sections at room temperature for 10 min, followed by incubation with the TUNEL reaction mixture for 1 h. Subsequently, slides were incubated with DAPI solution (G1012, Servicebio) for 10 min in the dark. Finally, the sections were mounted in an anti-fade mounting medium (G1401, Servicebio). The sections were then examined with a NIKON ECLIPSE E100 microscope (Nikon), and images were captured and analyzed using a NIKON DS-U3 imaging system (Nikon).

### RNA sequencing and data analysis

RNA sequencing (RNA-seq) and bioinformatics analysis were conducted by Gene Denovo Biotechnology (Guangzhou, China). Total RNA was extracted from samples using TRIzol reagent (Invitrogen, Waltham, CA, USA). RNA concentration, purity (OD260/280 ≥ 1.8, OD260/230 ≥ 2.0), and integrity (RNA Integrity Number (RIN) ≥ 7.0) were assessed using a NanoDrop 2000 Spectrophotometer (Thermo Fisher Scientific) and an Agilent 2100 Bioanalyzer (Agilent Technologies, Santa Clara, CA, USA). Subsequently, the enriched mRNA was fragmented into short segments using a fragmentation buffer and then reverse transcribed into cDNA utilizing the NEBNext® Ultra™ II FS DNA Library Prep Kit for Illumina (New England Biolabs; Ipswich, MA, USA). The libraries were quality-checked using the DNA 1000 Assay Kit (Agilent Technologies) and subsequently sequenced on the Illumina NovaSeq 6000 platform (Illumina, San Diego, CA, USA). After quality control had been performed on the raw data with FastQC (https://www.bioinformatics.babraham.ac.uk/projects/fastqc/), reads were aligned to the reference genome (GRCh38; https://www.ncbi.nlm.nih.gov/assembly/GCF_000001405.39/) by using Hisat2 (Kim et al. 2015). Transcript assembly and quantification were carried out with StringTie (Pertea et al. 2015), and differentially expressed genes (DEGs) were identified using DESeq2 (Love et al. 2014) by applying the criteria |log2FC|≥ 1 and false discovery rate (FDR) < 0.05. Functional annotation and enrichment analysis of DEGs were performed utilizing the Kyoto Encyclopedia of Genes and Genomes (KEGG; https://www.genome.jp/kegg/), Gene Ontology (GO; http://geneontology.org/) databases, and Gene Set Enrichment Analysis (GSEA)(Subramanian et al. 2005).

### Detection of steroid hormones

QTRAP 6500 Liquid Chromatography Tandem Mass Spectrometry (LC–MS/MS) Systems (SCIEX, Toronto, Canada) were employed to quantify steroid hormones in both cell cultures and mouse serum. Four distinct steroid hormone detection kits were provided by Metware Biotechnology (Wuhan, China). For sample preparation, pre-processed samples were utilized, with additional processing being required for cell samples. These cell samples were homogenized using a grinder, followed by extraction with methanol via vortexing and centrifugation. The resulting extracts were concentrated at 20 °C, reconstituted in methanol, and subjected to further vortexing and centrifugation. Finally, the supernatant was collected for subsequent LC–MS/MS analysis.

### Statistical analysis

All bar charts were generated using GraphPad Prism 8.0 (GraphPad Software, San Diego, CA, USA). Data were presented as means ± standard deviation (SD). Statistical significance between groups was assessed using a two-tailed t-test. A *p*-value of < 0.05 (*) was considered to indicate a significant difference between groups, with *p* < 0.01 (**) denoting a highly significant difference, and *p* < 0.001 (***) indicating an extremely significant difference.

## Results

### Celastrol affects ovarian development in young mice

To investigate the effects of celastrol on ovarian development, we utilized a study model of young mice. Celastrol was administered to 3-week-old mice via intraperitoneal injection at various concentrations every other day for 3 weeks. During the 3-week treatment period with celastrol, although all mice experienced body weight gain due to growth/development, the body weights of mice in all treatment groups were lower than those of the control group. However, by the conclusion of the treatment, the body weights of both groups were comparable, with the control group averaging 16.78 g and the 3 mg/kg treatment group averaging 15.00 g. In contrast, the 6 mg/kg treatment group experienced a significant reduction in body weight, with a mean of 13.09 g, suggesting potential toxicity associated with this higher dosage.

**Fig. 1:**
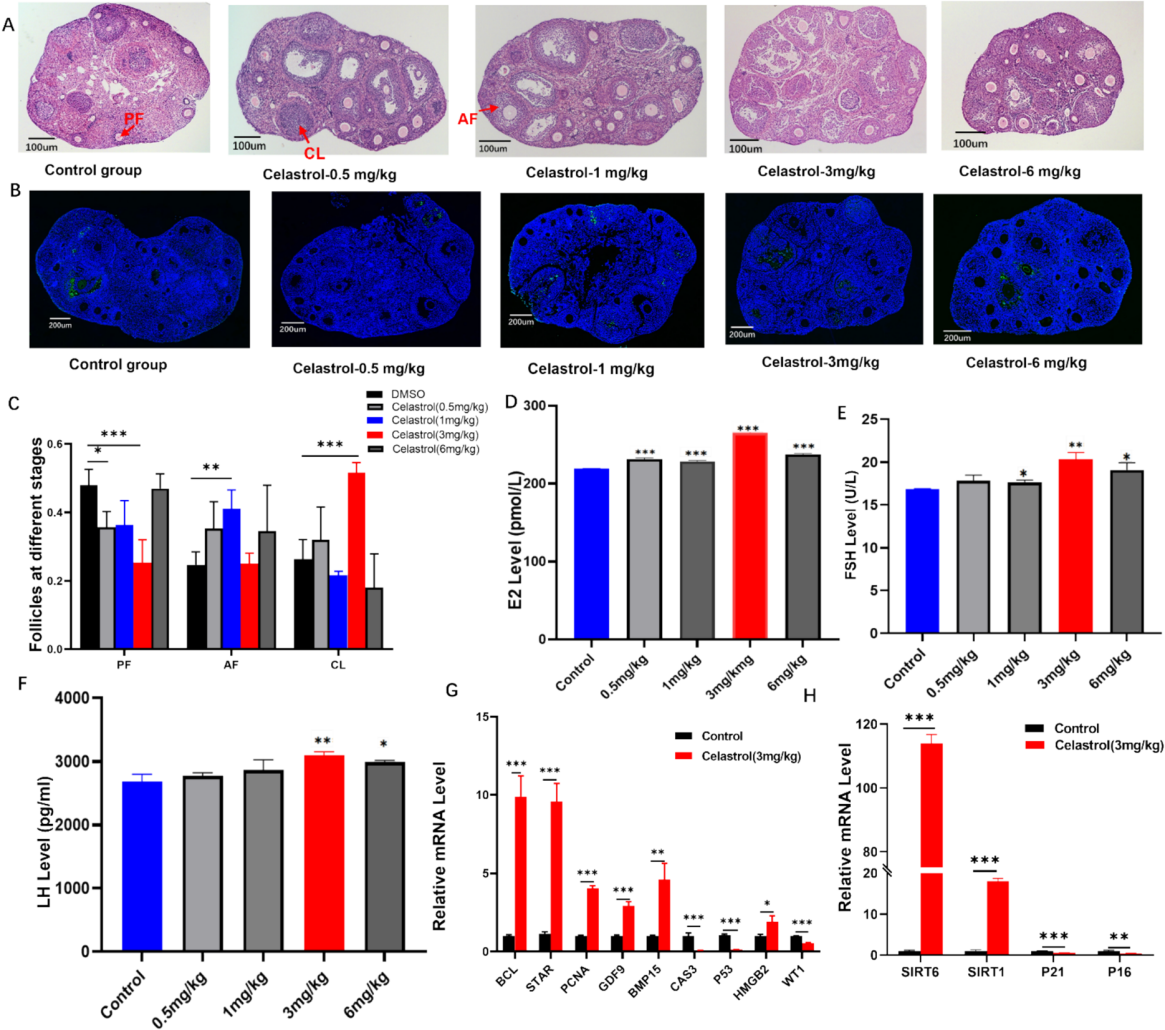
Effects of different concentrations of celastrol on ovarian development in mice. **A** H&E staining revealed the ovarian morphology in young mice treated with different concentrations of celastrol (*n* = 8 per group). Arrows indicate the presence of follicular structures, including CL, AF, PF. Scale bar: 100 μm. **B** The impact of celastrol on GC apoptosis in ovaries is illustrated, with green fluorescence highlighting apoptotic cells. Scale bar: 200 μm. **C** Quantification of PF, AF, and CL in murine ovaries following celastrol treatment. **D**–**F** The effects of celastrol on serum levels of E2, FSH, and LH measured by ELISA. **G** mRNA expression levels of genes associated with ovarian development measured by RT-PCR. **H** mRNA expression levels of genes related to ovarian aging measured by RT-PCR. AF: antral follicle; BCL: B-cell lymphoma; CASP3: caspase-3 (cysteine-aspartic protease 3); CL: corpus luteum; E2: estradiol; FSH: follicle-stimulating hormone; GDF9: growth differentiation factor 9; HMGB2: high mobility group box 2; LH: luteinizing hormone; PCNA: proliferating cell nuclear antigen; PF: preantral follicle; SIRT1: Sirtuin 1; SIRT6: Sirtuin 6; STAR: steroidogenic acute regulatory protein; WT1: Wilms’tumor 1

At the conclusion of the treatment period, the mice were euthanized for the collection of blood and ovarian tissues. H&E staining and TUNEL assays were conducted to evaluate ovarian development (Fig. 1A- B) and Supplementary Fig. 1. The results indicated that, at a concentration of 1 mg/kg, celastrol significantly increased the number of AFs (*p* < 0.01). In contrast, at a concentration of 3 mg/kg, we observed a significant elevation in the number of corpora lutea (CLs; *p* < 0.001; Fig. 1C), which typically indicated an increase in ovulation. However, following treatment with celastrol, there was no significant difference in the total number of follicles across all experiment groups when compared to the control group, as illustrated in Supplementary Fig. 2.

All three doses (0.5 mg/kg, 1 mg/kg, and 3 mg/kg) were found to decrease the apoptosis rate of murine ovarian GCs when compared to the control group, while no significant difference was observed between the control group and the 6 mg/kg treated group (Fig. 1B and Supplementary Fig. 1). Serum levels of E2, FSH, and LH were measured, revealing that celastrol significantly increased estrogen-related hormone levels (Fig. 1D-F and Supplementary Fig. 3). Notably, at 3 mg/kg, the increase in estrogen levels was most pronounced when compared with that of the control group (*p* < 0.001; Fig. 1D).

Further analysis of the mRNA expression of the ovarian tissue demonstrated that celastrol at 3 mg/kg upregulated the expression of *BCL* (*p* < 0.001), *STAR* (*p* < 0.001) and *PCNA* (*p* < 0.001), both of which are associated with cell proliferation, and of *GDF9* (*p* < 0.001) and bone morphogenetic protein 15 (*BMP 15*; *p* < 0.01), both of which play crucial roles in oocyte development (Fig. 1G). Additionally, celastrol inhibited the expression of apoptosis-related genes, including that of *Casp3* (*p* < 0.001) and of *P53* (*p* < 0.001; Fig. 1G). Interestingly, celastrol was also associated with the regulation of aging. It upregulated the mRNA expression levels of longevity-related genes *SIRT6* (*p* < 0.001) and *SIRT1* (*p* < 0.001), while downregulating the expression of senescence-associated genes *P21* (*p* < 0.001) and *P16* (*p* < 0.001; Fig. 1 H). These findings suggest a potential role for celastrol in delaying ovarian aging.

### Celastrol promotes oocyte maturation and regulates GC proliferation/apoptosis

**Fig. 2:**
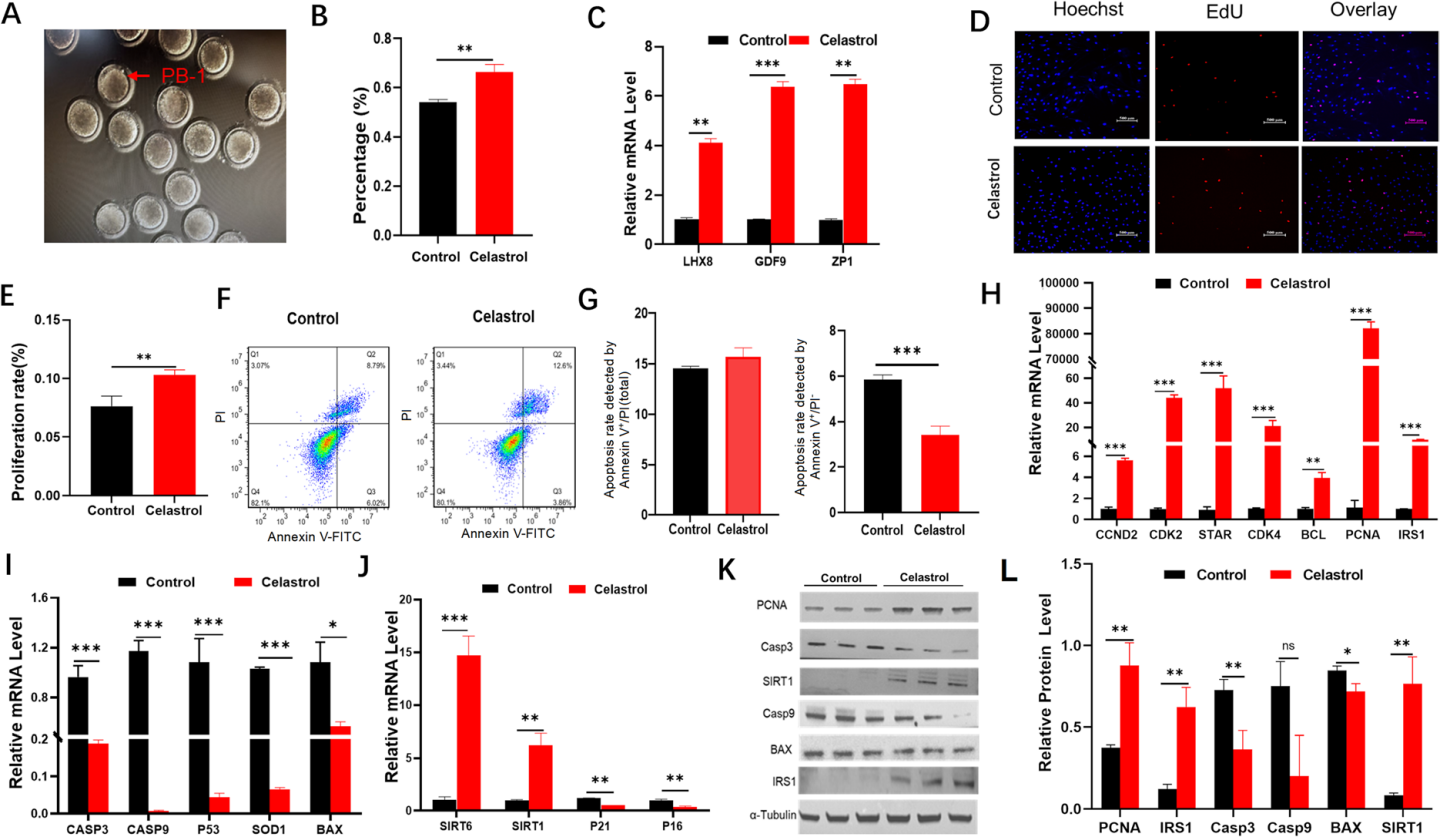
Effects of celastrol on porcine oocytes and GCs. **A**–**B** Impact of celastrol on the extrusion of the PB-1 in oocytes (*n* = 3). **C** mRNA expression levels of oocyte-related genes following treatment with celastrol. **D**–**E** Effect of celastrol on proliferation of GCs (*n* = 3). Scale bar: 500 μm. **F**–**G** Influence of celastrol on GC apoptosis (*n* = 3). **H**–**J** mRNA expression levels of genes associated with proliferation, apoptosis, and senescence in GCs after celastrol treatment. **K**–**L** Protein expression levels of proliferation-, apoptosis-, and senescence-related genes in GCs after celastrol treatment. BAX: Bcl-2-associated X protein; BCL: B-cell lymphoma; CASP3: Caspase-3 (cysteine-aspartic protease 3); CASP9: Caspase-9 (cysteine-aspartic protease 9); CDK2: cyclin-dependent kinase 2; CDK4: cyclin-dependent kinase 4; CCND2: cyclin D2; GDF9: growth differentiation factor 9; HMGB2: high mobility group box 2; IRS1: insulin receptor substrate 1; LHX8: LIM homeobox 8; PCNA: proliferating cell nuclear antigen; SIRT1: sirtuin 1; SIRT6: sirtuin 6; SOD1: superoxide dismutase 1; STAR: steroidogenic acute regulatory protein; ZP1: zona pellucida sperm-binding protein 1

To investigate the effects of celastrol on oocyte maturation, porcine oocytes were isolated from follicles measuring 3–5 mm in diameter and cultured in the in vitro maturation medium supplemented with 0.5 μM celastrol. After 48 h, the maturation rates of the oocytes were assessed. The results indicated that celastrol treatment significantly enhanced the extrusion of the PB-1 compared to the control group (DMSO; *p* < 0.01, Fig. 2A, B). Additionally, RT-PCR analysis revealed that celastrol significantly upregulated the mRNA expression of genes associated with oocyte maturation, including LIM Homeobox 8 (*LHX8*; *p* < 0.01) and growth differentiation factor 9 (*GDF9*; *p* < 0.001), and of a gene essential for zona pellucida function (*ZP1*; the gene for zona pellucida sperm-binding protein 1; *p* < 0.01, Fig. 2C).

To evaluate the impact of celastrol on GCs, porcine GCs were treated with 0.5 μM celastrol. This treatment significantly enhanced GC proliferation (*p* < 0.01, Fig. 2D, E) and increased the mRNA expression of proliferation-related genes, including *STAR* (*p* < 0.001), *BCL* (*p* < 0.01), and *PCNA* (*p* < 0.001; Fig. 2H). Furthermore, PCNA protein levels were significantly elevated (*p* < 0.01; Fig. 2K, L). In contrast, celastrol significantly reduced GC apoptosis (*p* < 0.01, Fig. 2F, G). The expression of apoptosis-related genes, including *Casp3* (*p* < 0.01), Bcl-2-associated X protein (*BAX*; *p* < 0.05), *Casp9* (*p* < 0.001), and *P53* (*p* < 0.001), was markedly downregulated at the mRNA level (Fig. 2I). Additionally, a notable decrease occurred in the protein expression of Casp3 (*p* < 0.01) and BAX (*p* < 0.05; Fig. 2K, L). Moreover, celastrol influenced the expression of genes associated with cellular aging. It significantly upregulated the mRNA levels of longevity-related genes *SIRT1* (*p* < 0.001) and *SIRT6* (*p* < 0.01) and the protein expression of *SIRT1* (*p* < 0.01; Fig. 2J, K). Concurrently, celastrol downregulated the mRNA expression of senescence-related genes *P21* (*p* < 0.01) and *P16* (*p* < 0.01; Fig. 2J, K).

In addition to its well-known anti-inflammatory and antioxidant properties, celastrol has been reported to modulate various signaling pathways, particularly the insulin/insulin receptor substrate pathway (Zhou et al. 2024). Consequently, we investigated the levels of IRS1, a crucial adapter protein in insulin signaling, following celastrol treatment. Our findings revealed that celastrol significantly increased both the mRNA (*p* < 0.001) and protein (*p* < 0.01) expression levels of *IRS1*, a key gene involved in the insulin signaling pathway (Fig. 2H, K).

### Effect of celastrol on ovarian aging of mice

**Fig. 3:**
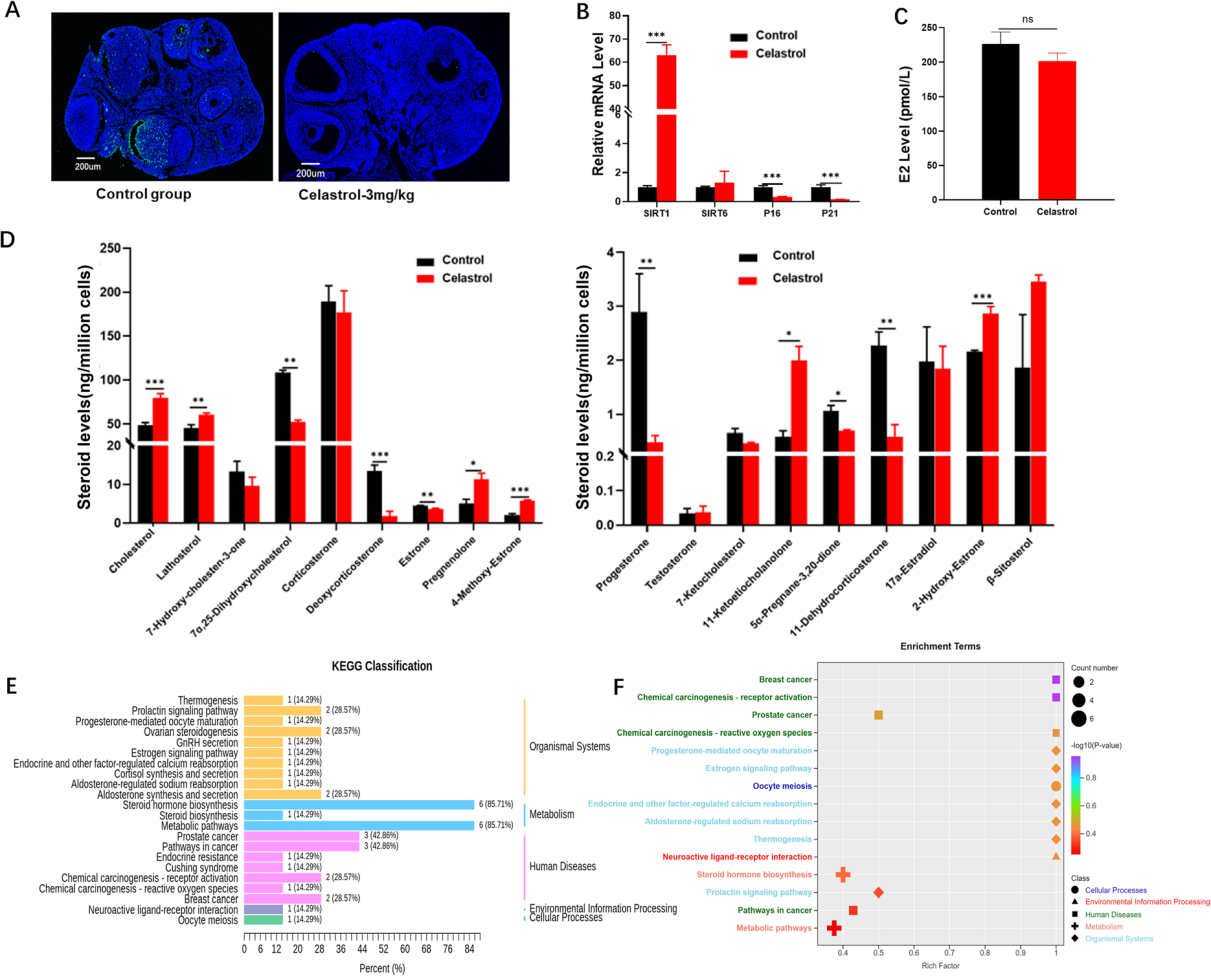
Effects of celastrol (3 mg/kg) on ovarian function in 12-month-old female mice. **A** TUNEL assay illustrating ovarian morphology and apoptosis (*n* = 5 per group). Scale bars: 200 μm. **B** Quantitative analysis of mRNA expression levels of senescence-related genes (*n* = 3) after celastrol treatment. **C** Measurement of E2 levels in mouse serum after celastrol treatment. **D** Assessment of steroid hormone levels after celastrol treatment. **E**–**F** KEGG pathway analysis highlighting steroid hormone-enriched signaling pathways. SIRT1: sirtuin 1; SIRT6: sirtuin 6

12-month-old (middle-aged) female mice were utilized to investigate the effects of celastrol on aging. Celastrol was administered via intraperitoneal injection every other day at a dose of 3 mg/kg for 3 weeks. Results from the TUNEL assay demonstrated that, consistent with findings observed in young mice, celastrol reduced apoptosis in ovarian GCs of the 12-month-old female mice (Fig. 3A and Supplementary Fig. 4). Additionally, treatment with celastrol significantly increased the mRNA expression level of *SIRT6* (p < 0.001), while decreasing the mRNA levels of *P16* (*p* < 0.001) and *P21* (*p* < 0.001; Fig. 3B).

Although the expression level of E2 did not show a significant difference between the celastrol-treated and control groups (Fig. 3C), steroid/steroid hormone analysis revealed that celastrol significantly elevated the levels of cholesterol (*p* < 0.001), lathosterol (*p* < 0.01), and β-Sitosterol, and of the estrogen metabolites 2-Hydroxy-Estrone (*p* < 0.001) and 4-Methoxy-Estrone (*p* < 0.001; Fig. 3D). KEGG enrichment analysis indicated that celastrol primarily influenced pathways related to steroid biosynthesis, ovarian steroidogenesis, and estrogen signaling in the 12-month-old female mice (Fig. 3E, F).

### Transcriptomic changes induced by celastrol in 12-month-old mice

**Fig. 4:**
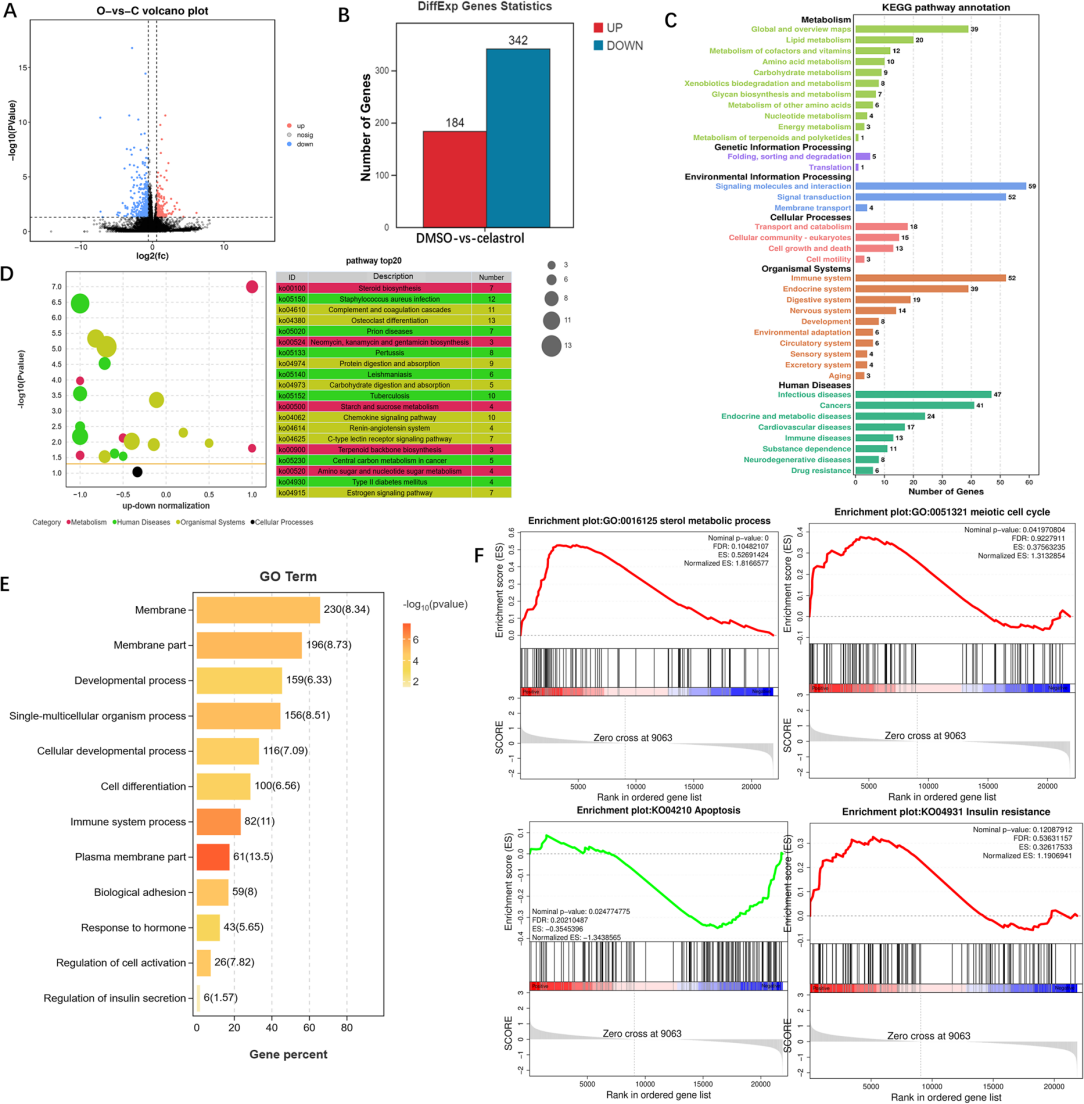
Transcriptomic changes induced by celastrol (3 mg/kg) in 12-month-old female mice with 3 weeks of treatment. **A** Volcano plot of DEGs in ovarian tissues from 12-month-old mice treated with celastrol and DMSO (control). **B** Bar chart illustrating the number of upregulated and downregulated DEGs. **C** KEGG functional annotation bar plot of DEGs. **D** KEGG enrichment bubble plot of DEGs. **E** GO pathway enrichment bar chart of DEGs across various pathways. **F** GSEA analysis of enriched gene sets, with blue and red bars indicating downregulated and upregulated genes, respectively

Transcriptome sequencing was then conducted to investigate the effects of celastrol on 12-month-old mice. Celastrol (3 mg/kg) was administered to these mice via intraperitoneal injection, and ovarian tissues were subsequently collected for total RNA extraction after 3 weeks of treatment. The transcriptomic analysis revealed that, compared with the control group, 184 genes were upregulated, and 342 genes were downregulated in the treatment group (Fig. 4A-B). KEGG enrichment analysis indicated that these DEGs are associated with biological functions related to the immune system, cancer, and signal transduction (Fig. 4C). Further examination of these DEGs highlighted their involvement in cholesterol synthesis and the estrogen signaling pathway. Additionally, the results suggested a potential link between these DEGs and the pathogenesis of type 2 diabetes (Fig. 4D).

GO enrichment analysis demonstrated that celastrol influenced various cellular processes, including cell membrane function, signal molecule transduction, insulin secretion, and cell development (Fig. 4E). GSEA further revealed that celastrol played a significant role in steroid biosynthesis, meiosis, and apoptosis (Fig. 4F). Notably, celastrol was also found to affect the insulin resistance pathway. In view of our previous study (Yuan et al. 2023) and the observed changes in IRS1 expression in response to celastrol treatment (Fig. 2K), these findings suggest that celastrol regulates follicular development through the modulation of IRS1.

### IRS1 influences ovarian development in young mice

**Fig. 5:**
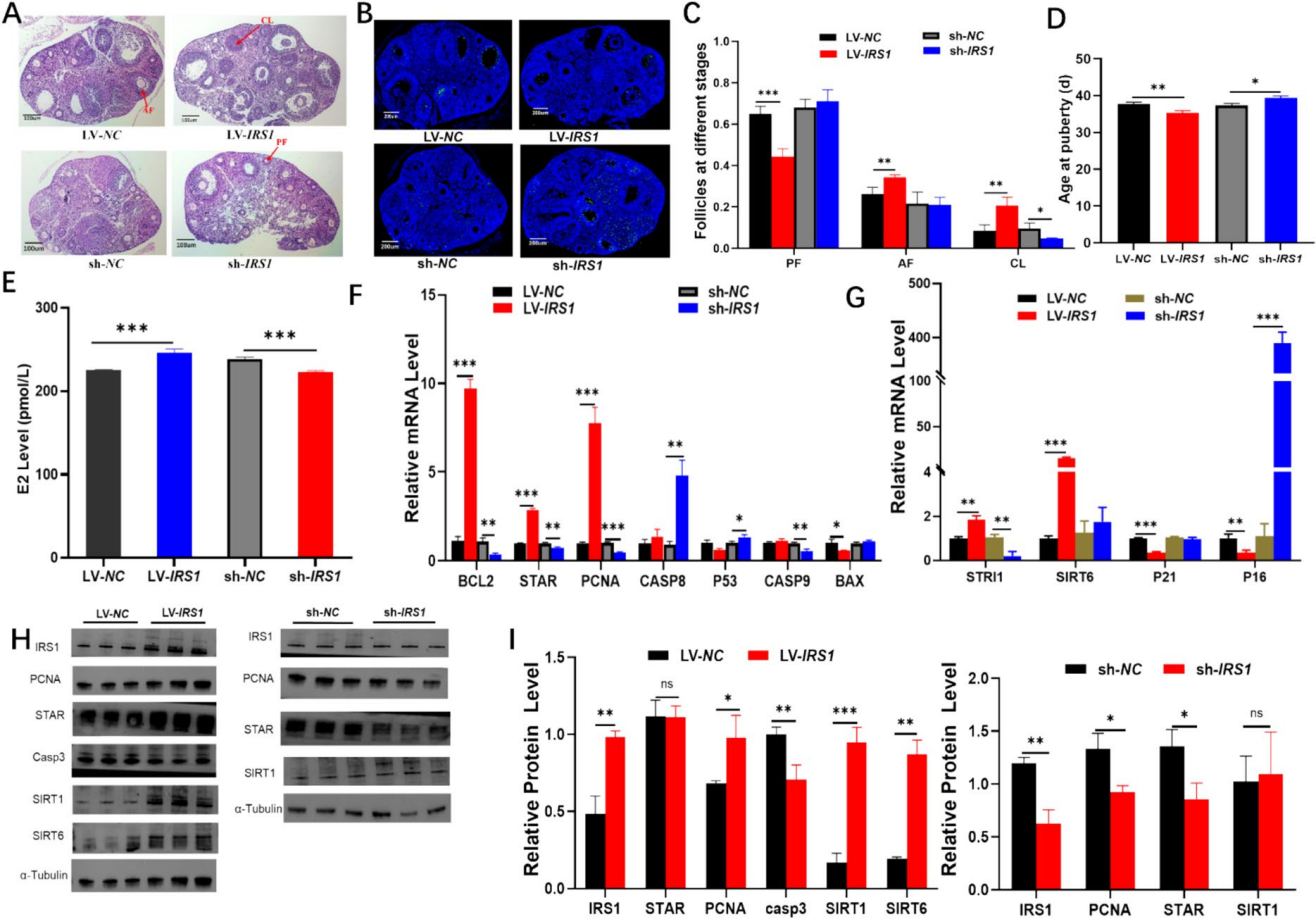
Effects of IRS1 on ovarian development in mice. **A** H&E staining analysis illustrating follicular development in mouse ovaries (*n* = 8 per group). Scale bar: 100 μm. **B** TUNEL assay to assess GC apoptosis in ovarian tissues. Scale bar: 200 μm. **C** Quantification of follicles at different developmental stages in the ovaries. **D** Effects of IRS1 on the age of vaginal opening in mice. **F**-**G** Quantitative analysis of mRNA expression levels of related genes in ovarian tissues. **H**-**I** Measurement of protein expression levels of associated genes in ovarian tissues. AF: antral follicle; BAX: BCL-2-associated X protein; BCL2: B-cell lymphoma 2; CASP3: Caspase-3 (cysteine-aspartic protease 3); CASP8: Caspase-8 (cysteine-aspartic protease 8); CASP9: Caspase-9 (cysteine-aspartic protease 9); CL: corpus luteum; IRS1: insulin receptor substrate 1; *LV-IRS1*: Lentiviral Vector-IRS1 overexpression; *LV-NC*: Lentiviral Vector-Negative Control; PCNA: proliferating cell nuclear antigen; PF: preantral follicle; *sh-IRS1*: short hairpin RNA -mediated IRS1 knockdown; *sh-NC*: shRNA-Negative Control; SIRT1: sirtuin 1; SIRT6: sirtuin 6; STAR: steroidogenic acute regulatory protein

Building on our previous observations of elevated IRS1 levels following treatment (Fig. 2H, K; as determined by RT-PCR and Western blot analysis) and the identification of insulin signaling-related pathways (Fig. 4D-F; from transcriptomic analysis), we hypothesized that IRS1, a key adapter protein in insulin signaling, might play an essential role in the celastrol-induced anti-aging effects on the ovary. To investigate this further, we intraperitoneally injected young female mice with synthesized lentiviral particles containing either *LV-IRS1*, *LV-NC* (negative control), *sh-IRS1* (short hairpin RNA targeting IRS1), or *sh-NC* (negative control for shRNA). Histological examination through H&E staining revealed that the *LV-IRS1* group exhibited a significant increase in the number of AFs and CLs in the ovaries compared with the control group (*LV-NC*), indicating that IRS1 promoted AF formation (*p* < 0.01) and oocyte ovulation (*p* < 0.01; Fig. 5A, C). In contrast, the sh-*IRS1* group showed a marked arrest in follicular development at the preantral stage compared with the *sh-NC* group (*p* < 0.001), suggesting that *IRS1* knockdown inhibited follicular development. TUNEL assays demonstrated that *LV-IRS1* treatment effectively reduced GC apoptosis in ovaries, whereas *sh-IRS1* treatment led to increased apoptosis of GCs (Fig. 5B and Supplementary Fig. 5).

Furthermore, the age of vaginal opening (an important indicator of sexual maturation) in the *LV-IRS1* group (35.3 ± 1.2 days) was significantly earlier than that in the control group (37.7 ± 1.2 days; *p* < 0.01). Conversely, vaginal opening in the *sh-IRS1* group (39.3 ± 0.58 days) was significantly delayed compared with that in the control group (37.3 ± 1.2 days; *p* < 0.05, Fig. 5D). Serum estrogen levels were significantly elevated in the *LV-IRS1* group compared with the control group (*p* < 0.001), whereas estrogen levels were notably reduced in the *sh-IRS1* group (*p* < 0.001; Fig. 5E).

Gene expression analysis of ovarian tissues revealed that, in the *LV-IRS1* group, mRNA levels of proliferation-related genes B-cell leukemia/lymphoma 2 (*BCL2*; *p* < 0.001), *STAR* (*p* < 0.001), and *PCNA* (*p* < 0.001) were significantly upregulated (Fig. 5F), accompanied by an increase in PCNA protein levels (*p* < 0.05; Fig. 5I). Conversely, the mRNA expression of the apoptosis-related gene *BAX* was downregulated (*p* < 0.01; Fig. 5F), together with decreased protein levels of CASP3 (*p* < 0.01; Fig. 5I). In the *sh-IRS1* group, a significant reduction was noted in mRNA levels of *BCL2* (*p* < 0.01), *STAR* (*p* < 0.01), and *PCNA* (*p* < 0.001), whereas the expression of apoptosis-related genes *CASP8* (*p* < 0.01), *CASP9* (*p* < 0.01), and *P53* (*p* < 0.05) was significantly upregulated.

Additionally, IRS1 influenced the expression of aging-related genes. Compared with the control group, the *LV-IRS1* group exhibited significantly increased mRNA (Fig. 5G) and protein levels (Fig. 5I) of *SIRT1* (*p* < 0.01) and *SIRT6* (*p* < 0.001), whereas the mRNA levels of *P21* (*p* < 0.001) and *P16* (*p* < 0.01) were downregulated (Fig. 5G).

## IRS1 influences oocyte maturation and steroid hormone levels

**Fig. 6:**
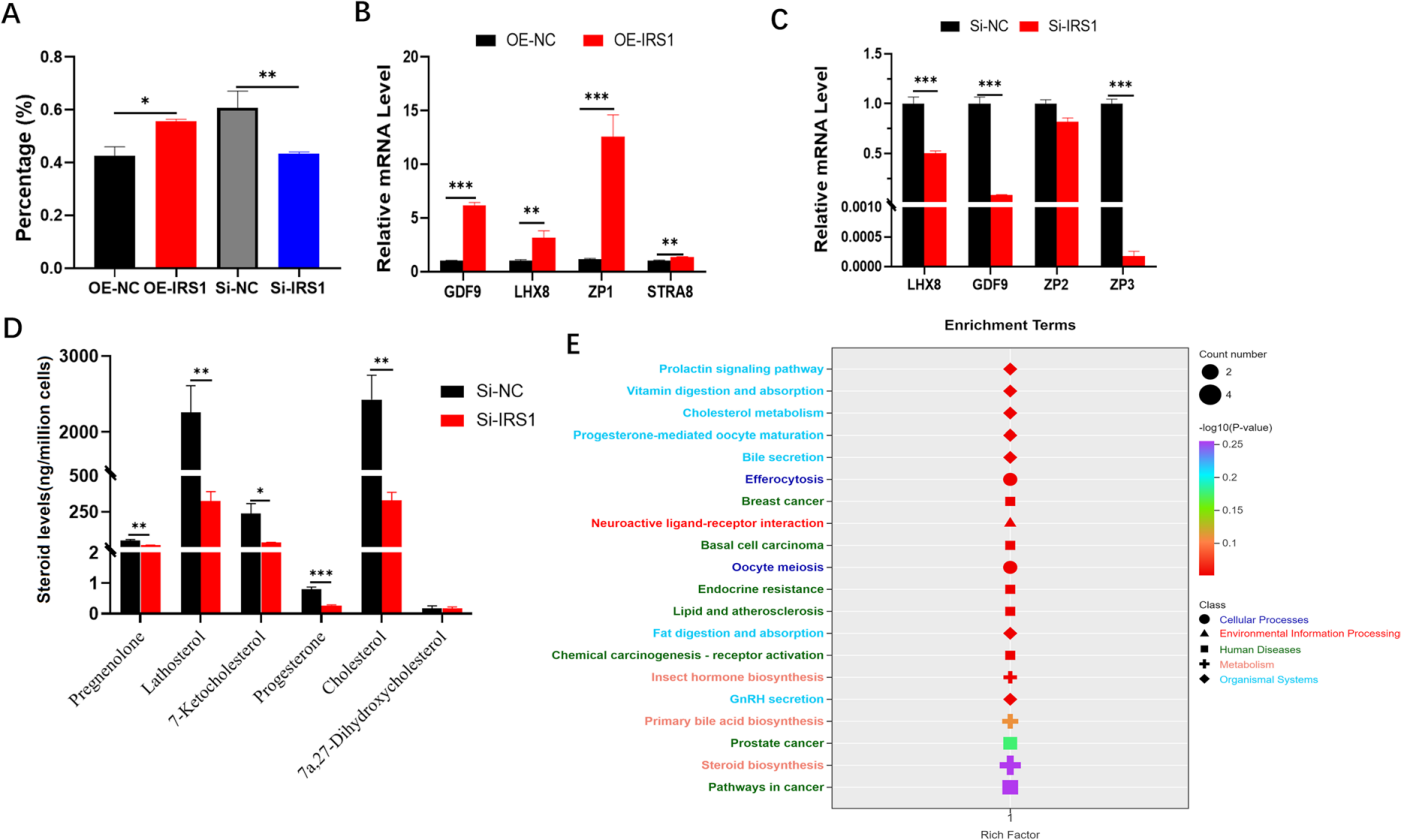
Effects of IRS1 on oocyte maturation and steroid hormone changes in porcine GCs. **A** Porcine oocyte maturation rate after in vitro maturation culture. **B-C** Changes in mRNA expression levels of oocyte-related genes after small RNA injection. **D** Alterations in steroid hormone levels in GCs following Si-RNA transfection. **E** KEGG pathway analysis of signaling pathways enriched in steroid hormone biosynthesis. GDF9: growth differentiation factor 9; LHX8: LIM homeobox 8; *OE-IRS1*: Overexpression Insulin Receptor Substrate 1; *OE-NC*: Overexpression-Negative Control; PCNA: proliferating cell nuclear antigen; *Si-IRS1*: Small Interfering RNA-Insulin Receptor Substrate 1; *Si-NC*: Small Interfering RNA-Negative Control; STRA8: Stimulated by retinoic acid 8; ZP1: zona pellucida sperm-binding protein 1; ZP2: zona pellucida sperm-binding protein 2; ZP3: zona pellucida sperm-binding protein 3

To investigate the effect of IRS1 on porcine oocytes, in vitro synthesized mRNA fragments of *OE-IRS1*, *Si-NC*, and *Si-IRS1* were microinjected into the cytoplasm of porcine oocytes, whereas the *OE-NC* were microinjected with sterile water. After 48 h, the number of oocytes that extruded PB-1 was quantified. The results showed that, compared with the control group, oocytes injected with *IRS1* exhibited a significantly higher maturation rate (*p* < 0.05), whereas oocytes injected with *si-IRS1* displayed a markedly reduced maturation rate (*p* < 0.01, Fig. 6A).

Quantitative analysis of collected oocytes revealed that, in the *IRS1*-injected group, the mRNA expression levels of *GDF9* (*p* < 0.001), *LHX8* (*p* < 0.01), *ZP1* (*p* < 0.001), and stimulated by retinoic acid 8 (*STRA8*; *p* < 0.01) were significantly upregulated (Fig. 6B). In contrast, oocytes injected with *si-IRS1* exhibited decreased mRNA expression levels of *GDF9* (*p* < 0.001), *LHX8* (*p* < 0.001), and Zona pellucida sperm-binding protein 2 (*ZP2*; *p* < 0.001; Fig. 6C).

Steroid analysis results indicated that GCs transfected with *si-IRS1* had decreased levels of steroid hormones, including lathosterol, cholesterol, and 7-ketocholesterol, and reduced levels of progesterone and pregnenolone (Fig. 6D). KEGG pathway analysis further demonstrated that these hormones were enriched in pathways related to ovarian steroid hormone biosynthesis and oocyte maturation (Fig. 6E).

### IRS1 influences transcriptomic changes in oocytes

**Fig. 7:**
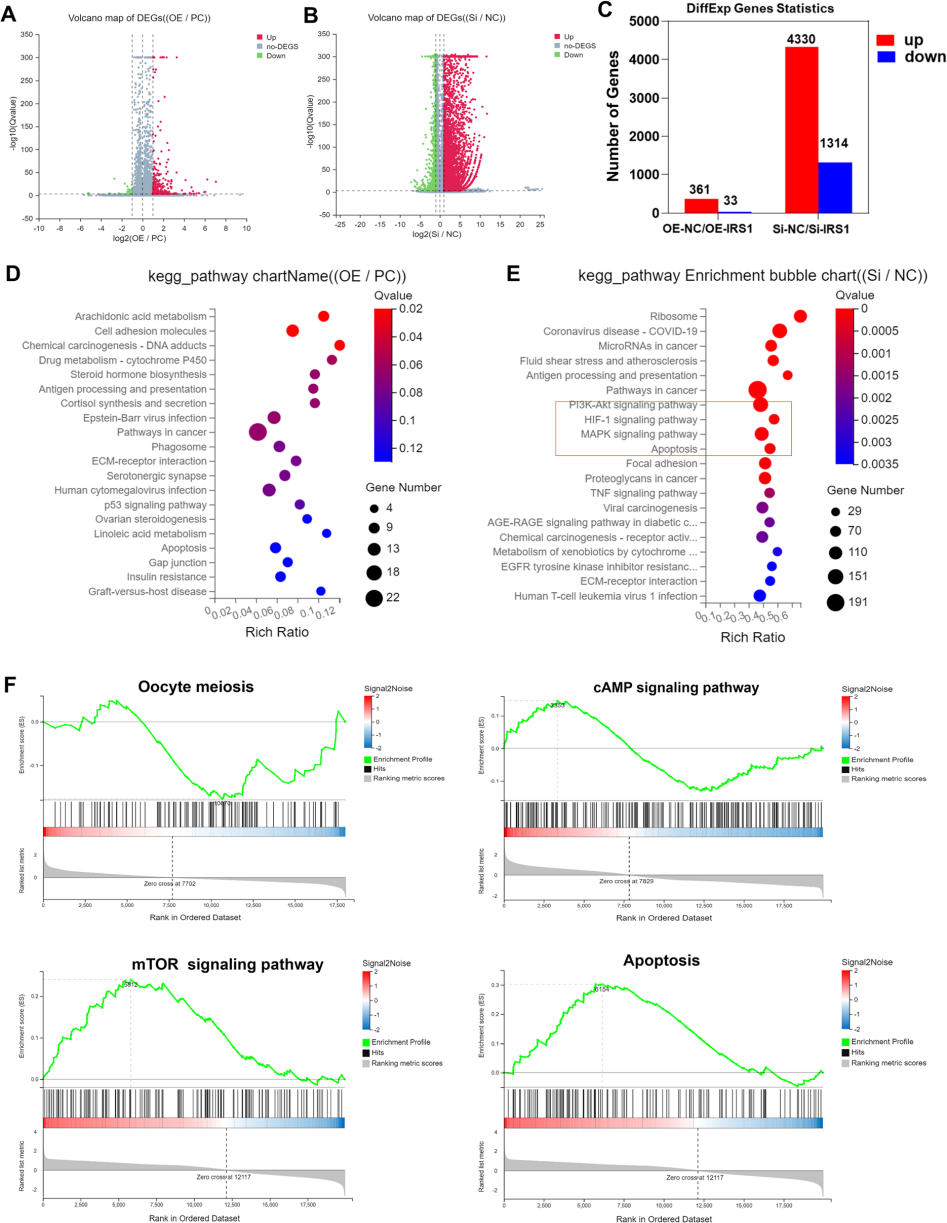
IRS1 influences transcriptomic changes in porcine oocytes. **A** Volcano plot of DEGs between the *IRS1* mRNA-injected group and the control group. **B** Volcano plot of DEGs between the si-RNA-injected group and the control group. **C** Bar chart showing the number of DEGs in both treatment groups compared with the control group. **D**-**E** KEGG pathway analysis of enriched signaling pathways associated with DEGs. **F** GSEA enrichment analysis of signaling pathways associated with DEGs

Single-cell transcriptomic analysis is a powerful technique that allows researchers to examine gene expression at the individual cell level (Kulkarni et al. 2019). To investigate the effects of IRS1 on the porcine oocyte transcriptome, in vitro synthesized mRNA fragments of *OE-IRS1*, *Si-NC*, and *Si-IRS1* were microinjected into the cytoplasm of porcine oocytes, with *OE-NC* receiving sterile water. After 24 h, oocytes were collected for transcriptome analysis; the results are shown in Fig. 7.

The transcriptomic results showed that, compared with the control group, 361 genes were upregulated, and 33 genes were downregulated in the *OE-IRS1* group. In contrast, in the *Si-IRS1* group, 4,330 genes were upregulated, and 1,314 genes were downregulated compared with the control group (Fig. 7C). KEGG enrichment analysis indicated that *IRS1* seemed to influence ribosome function (Fig. 7D). Additionally, *IRS1* was involved in the PI3K-AKT signaling pathway, hypoxia-inducible factor 1 signaling pathway, and MAPK signaling pathway (Fig. 7E). Further GSEA enrichment analysis suggested that *IRS1* regulated oocyte maturation by modulating the cyclic adenosine monophosphate (cAMP) signaling pathway and mTOR signaling pathway (Fig. 7F).

## Discussion

**Fig. 8:**
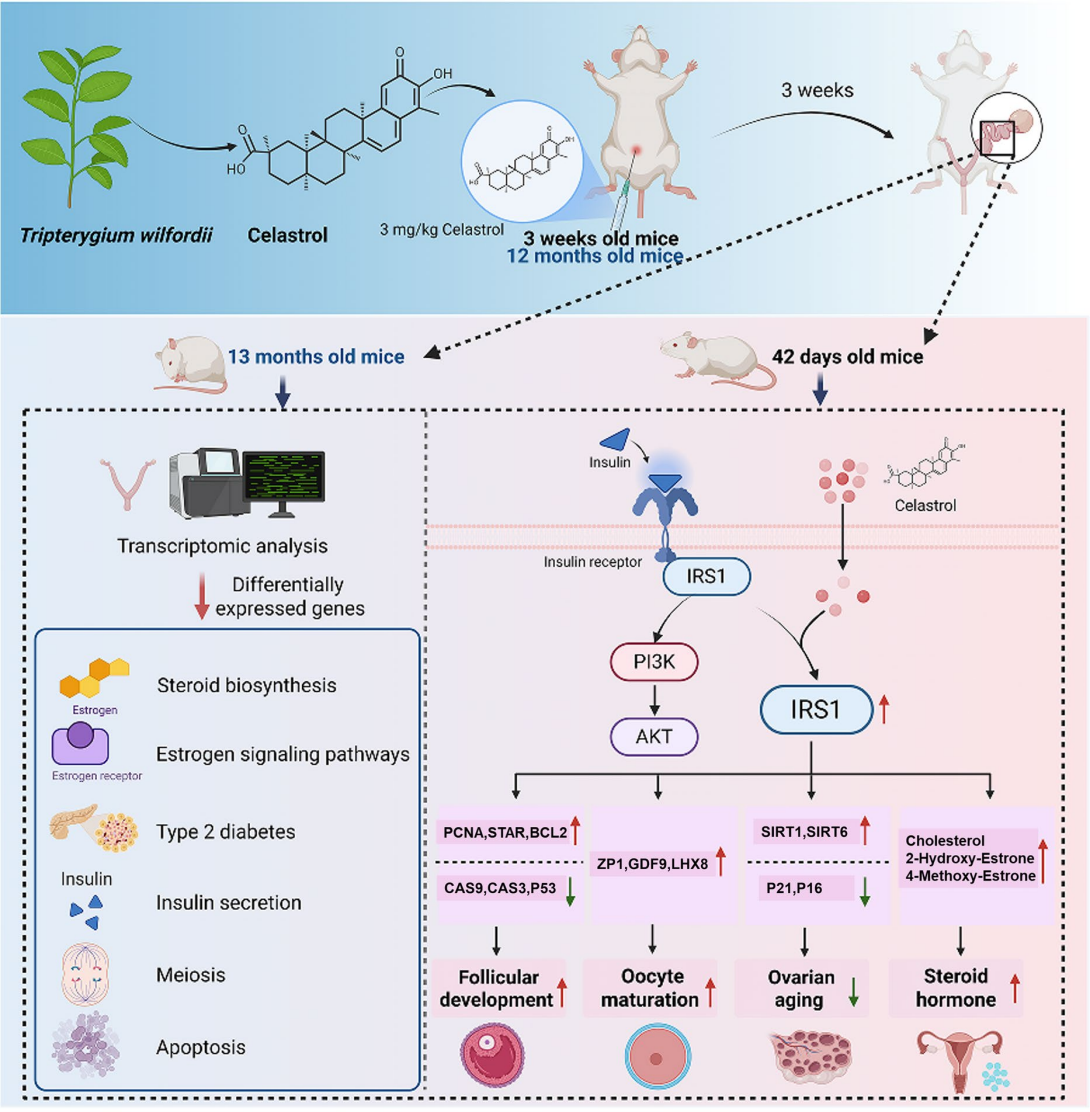
Celastrol enhances follicular development and alleviates ovarian aging by modulating IRS1 expression and its related pathways

Ovarian aging refers to the decline in ovarian function and fertility that occurs with age and can be categorized into two types: health aging and pathological aging (X. Wang et al. 2023). Healthy aging represents the natural process in which the ovaries gradually lose their functionality over time. In contrast, pathological aging encompasses abnormal changes in ovarian function that can result in various health issues or diseases. In this study, we employed female mouse models at two distinct ages, namely young (representing the reproductive starting phase) and 12 months (indicative of the ovarian decline phase), to rigorously investigate the effects of the natural compound celastrol on ovarian aging. By utilizing advanced methodologies such as in vitro microinjection, in vivo lentiviral vector-based gene overexpression/knockdown, and comprehensive single-cell transcriptomic analysis, we have elucidated the age-dependent regulatory effects of celastrol and its target, IRS1, and revealed their crucial roles in promoting follicular development and mitigating the adverse consequences of ovarian aging (Fig. 8).

### Celastrol and ovarian health/aging

Natural products have a long-standing history of use in the prevention and treatment of various diseases, offering numerous advantages (Atanasov et al. 2021). They typically contain a complex mixture of bioactive compounds that can enhance therapeutic effects while minimizing side effects compared with synthetic drugs. Many natural products are known for their anti-inflammatory, antioxidant, and antimicrobial properties, making them invaluable in managing chronic conditions such as diabetes, cardiovascular diseases, neurodegenerative diseases, infectious/inflammatory diseases, and cancer (Chaachouay & Zidane 2024; Goyal et al. 2024). One of the most renowned natural products is artemisinin (2015 Nobel Prize in Physiology or Medicine), together with its derivatives, which have demonstrated significant therapeutic effects against infections caused by parasites (e.g., malaria and Schistosoma), viruses, and bacteria, and against inflammatory/autoimmune diseases and skin disorders. This highlights the potential of natural compounds, not only as effective therapeutic agents, but also as crucial components in holistic approaches to health and disease management (Chung et al. 2022; Huang et al. 2023).

Celastrol is a natural compound recognized for its diverse pharmacological properties, including anti-inflammatory, anti-tumor, neuroprotective, and metabolic disease-related effects (Paris et al. 2010; Song et al. 2023; Xiao et al. 2021). Despite its broad therapeutic potential, there is a notable paucity of research examining its impact on ovarian health and associated disorders. In this study, we investigated the anti-aging effects of celastrol using both in vivo and in vitro models. Our findings have revealed that celastrol positively influences oocyte maturation and regulates GC proliferation/apoptosis, thereby promoting ovarian development and delaying the aging process of the ovaries. Interestingly, our results stand in contrast to a previous study that suggested celastrol, even at a dosage of 1 mg/kg, could induce premature ovarian insufficiency, potentially jeopardizing reproductive health and accelerating ovarian aging by disrupting critical cellular processes (Wen et al. 2023).

Our findings have also shown that celastrol significantly increases the blood levels of FSH and LH (Fig. 1E–F). Notably, celastrol has been identified as a leptin sensitizer, substantially enhancing leptin sensitivity in obese mice and leading to reduced food intake and improved body weight regulation (Liu et al. 2015). The mechanism underlying this action primarily involves the inhibition of protein tyrosine phosphatases in the hypothalamus, which serve as negative regulators of leptin signaling (Pfuhlmann et al. 2018). Furthermore, celastrol alleviates endoplasmic reticulum stress in the hypothalamus, further contributing to its metabolic benefits (He et al. 2021).

Although the direct evidence regarding celastrol’s effects on the pituitary gland is limited, the close relationship between the hypothalamus and pituitary gland in regulating various physiological processes (including metabolism, energy balance, and hormone regulation) suggests that indirect effects might occur. Therefore, we considered it reasonable to hypothesize that celastrol influences the secretion of FSH, LH, and other hormones, such as growth hormone, oxytocin, and thyroid-stimulating hormone, all of which play vital roles in ovarian function and aging (Colella et al. 2021).

In the present study, we observed that elevated cholesterol levels and DEGs underscore their involvement in cholesterol synthesis following celastrol treatment. Cholesterol serves as a crucial precursor for the synthesis of steroid hormones, including estrogen and progesterone. Maintaining adequate cholesterol levels is essential for the proper functioning of the ovaries, as these hormones play a vital role in regulating the menstrual cycle, ovulation, and overall fertility (Huang et al. 2019). As individuals age, ovarian function tends to decline, often resulting in decreased hormone production and altered cholesterol metabolism. This decline can contribute to various age-related health issues. Conversely, maintaining optimal cholesterol levels may support ovarian health and hormone synthesis, potentially alleviating some effects of aging and promoting reproductive longevity (Yang et al. 2022).

### IRS1 signaling and ovarian health/aging

Insulin receptors are present in various ovarian cell types, including GC, oocytes, and theca cells. Insulin signaling plays a crucial role in ovarian function/health, influencing various processes such as follicular development, hormone synthesis, and oocyte maturation (Dupont & Scaramuzzi 2016). In healthy ovaries, insulin facilitates the action of gonadotropins, promoting estrogen production and supporting reproductive health. However, insulin resistance can disrupt normal ovarian function, leading to irregular menstrual cycles, anovulation, and hyperandrogenism (Zhao et al. 2023). As women age, insulin sensitivity typically declines, contributing to metabolic disorders and exacerbating ovarian aging (Kolb et al. 2023). This decline in insulin sensitivity frequently results in elevated insulin levels, which may cause chronic inflammation and oxidative stress, further compromising ovarian health (Weinberg Sibony et al. 2024). Addressing insulin resistance through lifestyle modifications or pharmacological interventions is essential for improving ovarian function and mitigating age-related reproductive decline.

The key finding of this study is that celastrol treatment leads to a notable increase in IRS1 expression, and we have evidence indicating that IRS1 has independent pro-folliculogenesis activity. IRS1 is a central adaptor protein in the insulin/IGF-1 signaling pathway, and its expression level is closely associated with oocyte maturation capacity (Huangfu et al. 2023). In recent years, increasing attention has been directed toward targeting the IRS1 pathway with natural compounds for the treatment of various diseases, including diabetes (Yang et al. 2025; Yang et al. 2024), Alzheimer’s disease (K. X. Zhang et al. 2024), aging (Liu et al. 2025), and cancer (Mukundh et al. 2024). However, the application of IRS1-targeted therapeutics specifically in the context of ovarian development and aging is still in its early stages.

In this study, we have elucidated the beneficial effects of IRS1 and the underlying mechanisms driving these effects. First, IRS1 promotes follicular development by upregulating genes associated with cell proliferation, while simultaneously downregulating those linked to apoptosis and aging. Second, it facilitates oocyte maturation by enhancing the expression of key maturation-related genes such as *GDF9*, *LHX8*, *ZP1*, and *STRA8*, and by promoting steroid hormone biosynthesis. Third, IRS1 plays a crucial role in several essential biological processes, including the activation of the MAPK signaling pathway, steroid biosynthesis, and ribosomal function. Fourth, it regulates oocyte maturation through the modulation of the cAMP signaling pathway and various cell cycle processes.

### Mechanisms of celastrol′s action in ovarian health and aging

Evidence indicates that celastrol regulates insulin signaling through multiple mechanisms, alleviating insulin resistance and demonstrating therapeutic potential for diabetes management. Specifically, celastrol enhances insulin signaling by increasing the phosphorylation of key pathway proteins such as IRS1, AKT, and AS160 (Bhatia et al. 2019). Furthermore, as an inhibitor of nuclear factor kappa B (NF-κB), celastrol attenuates inflammatory responses, which in turn helps to reduce insulin resistance (Faheem et al. 2025). In the KsJ-lepr^*db*^/lepr^*db*^diabetic (*db/db*)mouse model, celastrol treatment significantly reduced fasting blood glucose and glycated hemoglobin levels, improved insulin sensitivity, and mitigated renal injury (Kim et al. 2013). In addition, celastrol modulated the expression of microRNAs that regulated insulin signaling. In a palmitate-induced insulin resistance model involving HepG2 cells, celastrol upregulated the expression of miR-223, restored the levels of glucose transporter type 4/IRS1, and thereby enhanced insulin signaling (Zhang et al. 2019). These findings suggest that celastrol exerts its biological effects, at least in part, through the modulation of the insulin signaling pathway.

In the present study, the upregulation of IRS1 expression was identified as a key feature of celastrol's mechanism of action. Celastrol might regulate IRS1 through several potential pathways. The first is epigenetic modification. Celastrol has been shown to inhibit histone deacetylase activity (S. Wang et al. 2023), which might enhance histone acetylation at the IRS1 promoter region, thereby promoting its transcription. The second is transcription factor activation. Celastrol can activate the AKT pathway (Gwag et al. 2013), and AKT activation leads to the phosphorylation of Forkhead Box O1(FOXO1) (Kim et al. 2008), which in turn inhibits its repressive activity on IRS1 expression. The third is protein stability regulation. As an inhibitor of heat shock protein 90 (HSP90) (Liew et al. 2022), celastrol might disrupt the interaction between HSP90 and IRS1, thereby reducing IRS1 ubiquitination and proteasomal degradation.

GCs function as central regulators of the follicular microenvironment and development, with their rate of apoptosis playing a crucial role in the progression of follicular atresia. Our study demonstrated that celastrol significantly inhibited GC apoptosis in both young and 12-month-old mice, an effect closely associated with increased IRS1 expression and the activation of SIRT1. Celastrol markedly upregulated SIRT1 expression in aged ovaries, and the cooperative role of SIRT1 further enhanced its anti-apoptotic effects. SIRT1 can suppress p53 transcriptional activity through deacetylation, and simultaneously promote the autophagic clearance of damaged mitochondria, thereby reducing ROS levels and attenuating apoptosis (Zhang et al. 2023). In addition, as a key adaptor protein in the insulin/IGF-1 signaling pathway, IRS1 regulates cell proliferation and apoptosis by activating the PI3K/AKT pathway (Cui & He 2022; Machado-Neto et al. 2011). The synergistic action of SIRT1 further reinforces this anti-apoptotic effect, highlighting the intricate interplay between these signaling pathways in the regulation of GC survival.

In aged ovaries, mitochondrial dysfunction caused by oxidative damage is a major trigger of GC apoptosis. The celastrol-induced upregulation of SIRT1 might ameliorate energy metabolism by enhancing peroxisome proliferator-activated receptor gamma coactivator 1 alpha (PGC-1α)-mediated mitochondrial biogenesis (Su et al. 2024). Moreover, SIRT1 and IRS1 exhibit reciprocal interactions in metabolic regulation: the IRS1–PI3K/AKT pathway can activate NAD kinase, leading to elevated NAD^+^ levels, which in turn promote SIRT1 activation (Hoxhaj et al. 2019). Conversely, SIRT1 has been reported to reduce the phosphorylation efficiency of IRS1, forming a positive feedback loop (Yoshizaki et al. 2009). This bidirectional regulatory mechanism might be the reason for celastrol being able to preserve GC function even in aged individuals.

Based on the findings of this study, the targeting of IRS1 for ovarian protection presents dual advantages. First, as an endogenous signaling hub, the activation of IRS1 can mimic physiological pro-folliculogenesis signals, thereby mitigating the side effects associated with exogenous hormonal interventions. Second, the multi-targeted nature of celastrol allows the simultaneous modulation of oxidative stress, inflammation, and metabolic dysregulation, all hallmarks of ovarian aging.

However, several challenges must be effectively overcome to facilitate future clinical translation. The first is the tissue-specific delivery. Systemic administration of celastrol might interfere with insulin sensitivity (Liu et al. 2024), necessitating the development of ovary-targeted nanocarriers (Gralewska et al. 2024). These targeted systems will possibly enhance the precision of drug delivery to the ovaries, minimizing systemic side effects. The second is the dose–response balance. High concentrations of celastrol might inhibit proteasome function and induce cytotoxicity (Xiao et al. 2024). To address this problem, comprehensive pharmacokinetic studies should be conducted to identify the effective concentration/dose of celastrol for treatment. The third is the long-term safety assessment. Whereas celastrol has shown promise in the treatment of various cancers, including ovarian cancer (C. Wang et al. 2023), the potential oncogenic risks associated with the sustained activation of IRS1 should be considered (Park et al. 2024; Reiss et al. 2012). Furthermore, the safety of celastrol during pregnancy, its effects on fetal and placental health, as well as its systemic toxicity and side effects (such as weight loss), require further exploration in future studies.

## Conclusion

Our study provides compelling evidence that celastrol may enhance ovarian development and preserve GC viability in both reproductively active and aging ovaries. These findings underscore the therapeutic potential of the celastrol-IRS1 axis in safeguarding female reproductive health and may offer a promising strategy for addressing some effects of ovarian aging. Furthermore, the modulation of IRS1 signaling and insulin signaling by natural compounds like celastrol could open new avenues for developing potential treatments and therapies for other aging/aging-related diseases and chronic conditions/diseases affecting other body systems.

## Supplementary Information

Below is the link to the electronic supplementary material.

Supplementary file 1 (PDF 360 KB)

## Data Availability

The raw RNA-seq data have been uploaded to the SRA database under submission numbers PRJNA1260748 and PRJNA1260756. These datasets will be made publicly available upon acceptance of the manuscript. Additionally, all other original data related to this study can be obtained from the corresponding author upon reasonable request.
