# Correction to: Celastrol modulates IRS1 expression to alleviate ovarian aging and to enhance follicular development

**DOI:** 10.1007/s10565-025-10107-6

**Published:** 2025-11-03

**Authors:** Yao Jiang, Yonghua Shi, Meng Lv, Tao Wang, Penghao Wang, Xiaolong Yuan, Fei Gao, Bin Ma

**Affiliations:** 1 School of Medical, Molecular and Forensic Sciences, Murdoch University, Murdoch, WA Australia; 2 Centre for Healthy Ageing, Health Futures Institute, Murdoch University, Murdoch, WA Australia; 3 State Key Laboratory of Swine and Poultry Breeding Industry, Guangdong Provincial Key Lab of Agro‑Animal Genomics and Molecular Breeding, College of Animal Science, Guangdong Laboratory of Lingnan Modern Agriculture, National Engineering Research Center for Breeding Swine Industry, South China Agricultural University, Guangzhou, Guangdong China; 4 Sun Yat‑Sen University Cancer Center, Sun Yat-Sen University, Zhongshan, Guangdong China; 5 The Kids Research Institute Australia, Nedlands, WA Australia; 6 Centre for Crop and Food Innovation, Food Future Institute, Murdoch University, Murdoch, WA Australia; 7 Shenzhen Branch, Guangdong Laboratory for Lingnan Modern Agriculture, Agricultural Genomics Institute at Shenzhen, Genome Analysis Laboratory of the Ministry of Agriculture, Chinese Academy of Agricultural Sciences, Shenzhen, Guangdong China

**Correction to: Cell Biol Toxicol (2025) 41:129**

10.1007/s10565-025-10079-7

Unfortunately, this article is posted online with errors in the main body text.

In page 2, 4^th^ paragraph under Introduction section, the year 2024a,b for reference W. Zhang should be changed to 2024.

In page 3, under Materials and methods section, the *C57BL/6 J* should be changed to *C57BL/6J*.

In the main body text, all occurrences mdash (—) should be changed to ndash (–).

In page 4, 2nd paragraph under Microinjection sub-section, catalog no. 747,720 should be changed to 747720.

In page 4, 1st paragraph under EdU proliferation assay sub-section, catalog no. 33,342 should be changed to 33342.

In the main body text, all occurrences of (’) should be changed to (′).

In page 5, Catalog no. 26,593-- should be changed to 26,593-.

In page 5, Catalog no. 10,205 should be changed to 10205.

In page 5, Catalog no. 17,509 should be changed to 17509.

In page 5, Catalog no. 66,470 should be changed to 66470.

In page 6, Catalog no. 13,423 should be changed to 13423.

In page 6, Catalog no. 10,380 should be changed to 10380.

In page 6, Catalog no. 10,883 should be changed to 10883.

In page 6, Catalog no. 13,161 should be changed to 13161.

In page 6, Catalog no. 13,572 should be changed to 13572.

In page 6, Catalog no. 11,224 should be changed to 11224.

In page 6, under Hematoxylin and eosin staining and TUNEL assay sub-section, Sects. should be changed to sections.

In page 12, Figure 4 legend, F) should be changed to F.

In page 15, 1st paragraph under Discussion section, the year 2023 a,b,c for reference X. Whang should be changed to 2023.

In page 15, 1st paragraph under Discussion section, the year 2024 a,b for reference K. X. Zhang should be changed to 2024.

In page 15, 1st paragraph under Discussion section, the year 2023 a,b,c for reference S. Wang should be changed to 2023.

In page 15, 1st paragraph under Discussion section, the year 2023 a,b,c for reference C. Wang et al. should be changed to 2023.

In page 20, Reference Atanasov et al. 2021, in the title the word Science, T. should be changed to Science Taskforce.

In page 21, Reference Bharath et al. 2020, the title “Metformin Enhances Autophagy and Normalizes Mitochondrial Function to Alleviate Aging-Associated Inflammation” should be set to sentence case as follows: “Metformin enhances autophagy and normalizes mitochondrial function to alleviate aging-associated inflammation.”

In page 21, Reference Feng et al. 2022, the title “LARS2 Regulates Apoptosis via ROS-Mediated Mitochondrial Dysfunction and Endoplasmic Reticulum Stress in Ovarian Granulosa Cells.” should be set to sentence case as follows: “LARS2 regulates apoptosis via ROS-mediated mitochondrial dysfunction and endoplasmic reticulum stress in ovarian granulosa cells.”

There are typos found in Figures 2, 3, 4, 6, 7 and 8. The corrected figures are shown below.


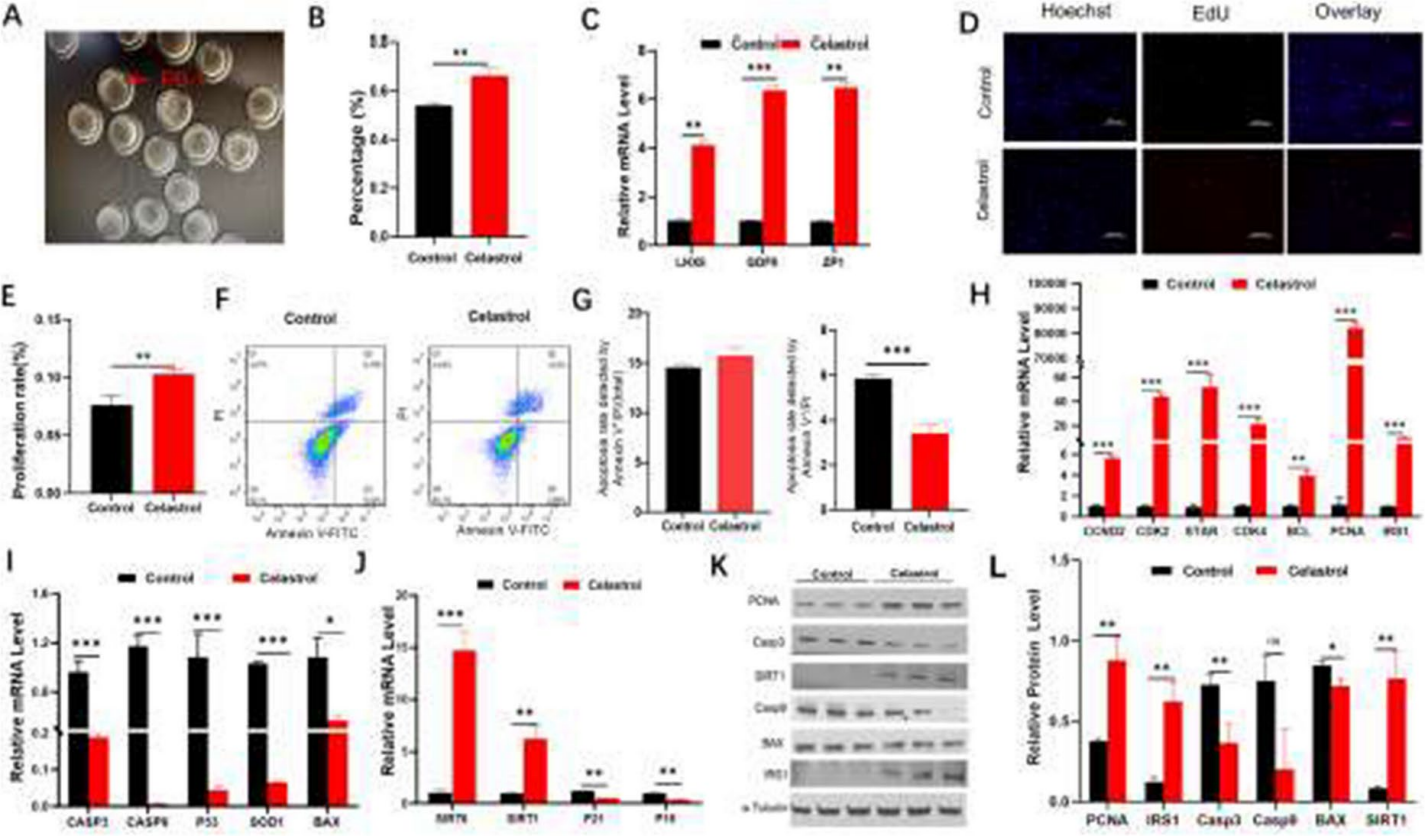


**Fig. 2** Effects of celastrol on porcine oocytes and GCs. **A**–**B** Impact of celastrol on the extrusion of the PB-1 in oocytes (*n* = 3). **C** mRNA expression levels of oocyte-related genes following treatment with celastrol. **D**–**E** Effect of celastrol on proliferation of GCs (*n* = 3). Scale bar: 500 μm. **F**–**G** Influence of celastrol on GC apoptosis (*n* = 3). H–J mRNA expression levels of genes associated with proliferation, apoptosis, and senescence in GCs after celastrol treatment. **K**–**L** Protein expression levels of proliferation-, apoptosis-, and senescence-related genes in GCs after celastrol treatment. BAX: Bcl-2-associated X protein; BCL: B-cell lymphoma; CASP3: Caspase-3 zcysteine-aspartic protease 3); CASP9: Caspase-9 (cysteineaspartic protease 9); CDK2: cyclin-dependent kinase 2; CDK4: cyclin-dependent kinase 4; CCND2: cyclin D2; GDF9: growth differentiation factor 9; HMGB2: high mobility group box 2; IRS1: insulin receptor substrate 1; LHX8: LIM homeobox 8; PCNA: proliferating cell nuclear antigen; SIRT1: sirtuin 1; SIRT6: sirtuin 6; SOD1: superoxide dismutase 1; STAR: steroidogenic acute regulatory protein; ZP1: zona pellucida spermbinding protein 1


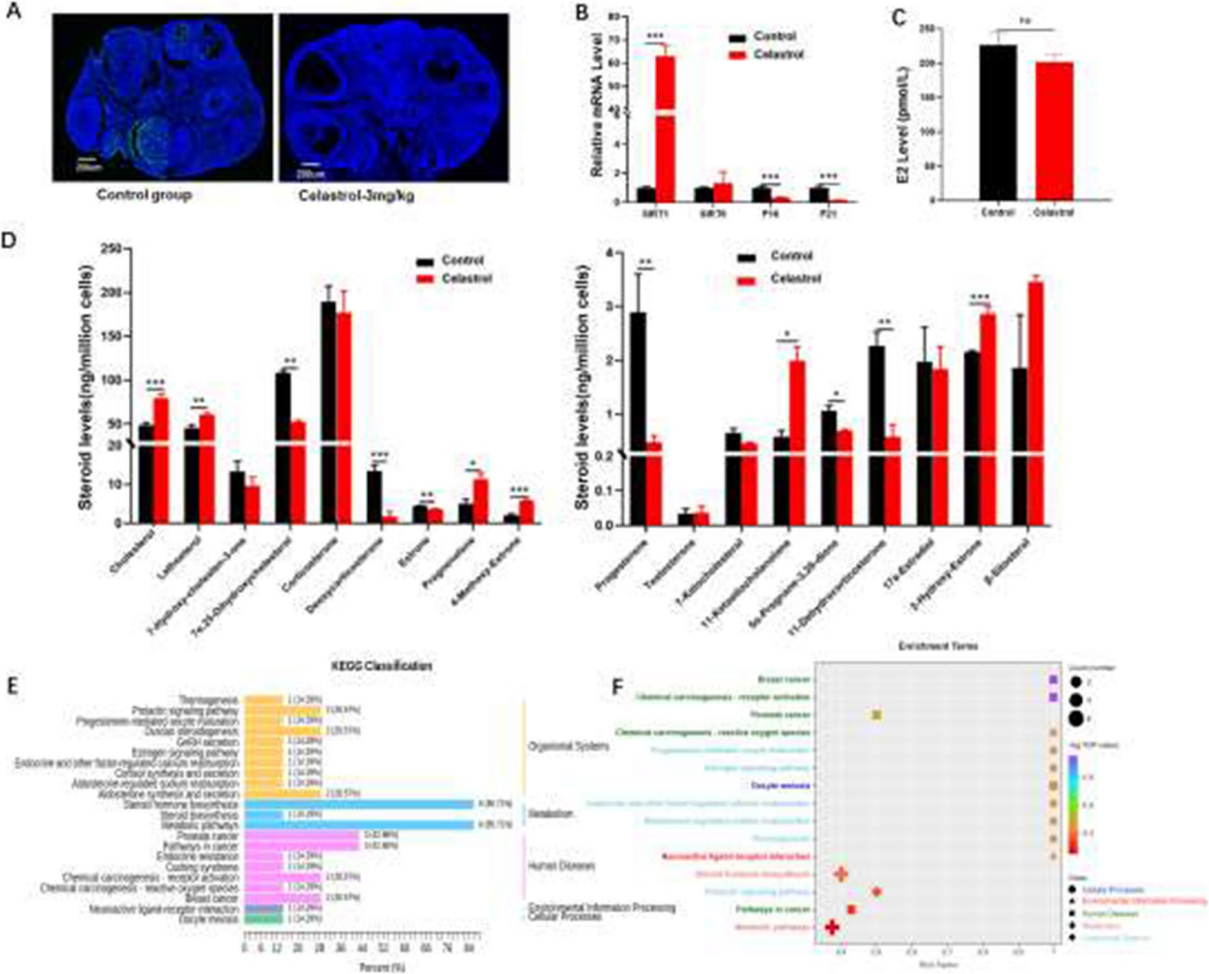


**Fig. 3** Effects of celastrol (3 mg/kg) on ovarian function in 12-month-old female mice. **A** TUNEL assay illustrating ovarian morphology and apoptosis (*n* = 5 per group). Scale bars: 200 μm. **B** Quantitative analysis of mRNA expression levels of senescence-related genes (*n* = 3) after celastrol treatment. **C** Measurement of E2 levels in mouse serum after celastrol treatment. **D** Assessment of steroid hormone levels after celastrol treatment. **E**–**F** KEGG pathway analysis highlighting steroid hormone-enriched signaling pathways. SIRT1: sirtuin 1; SIRT6: sirtuin 6


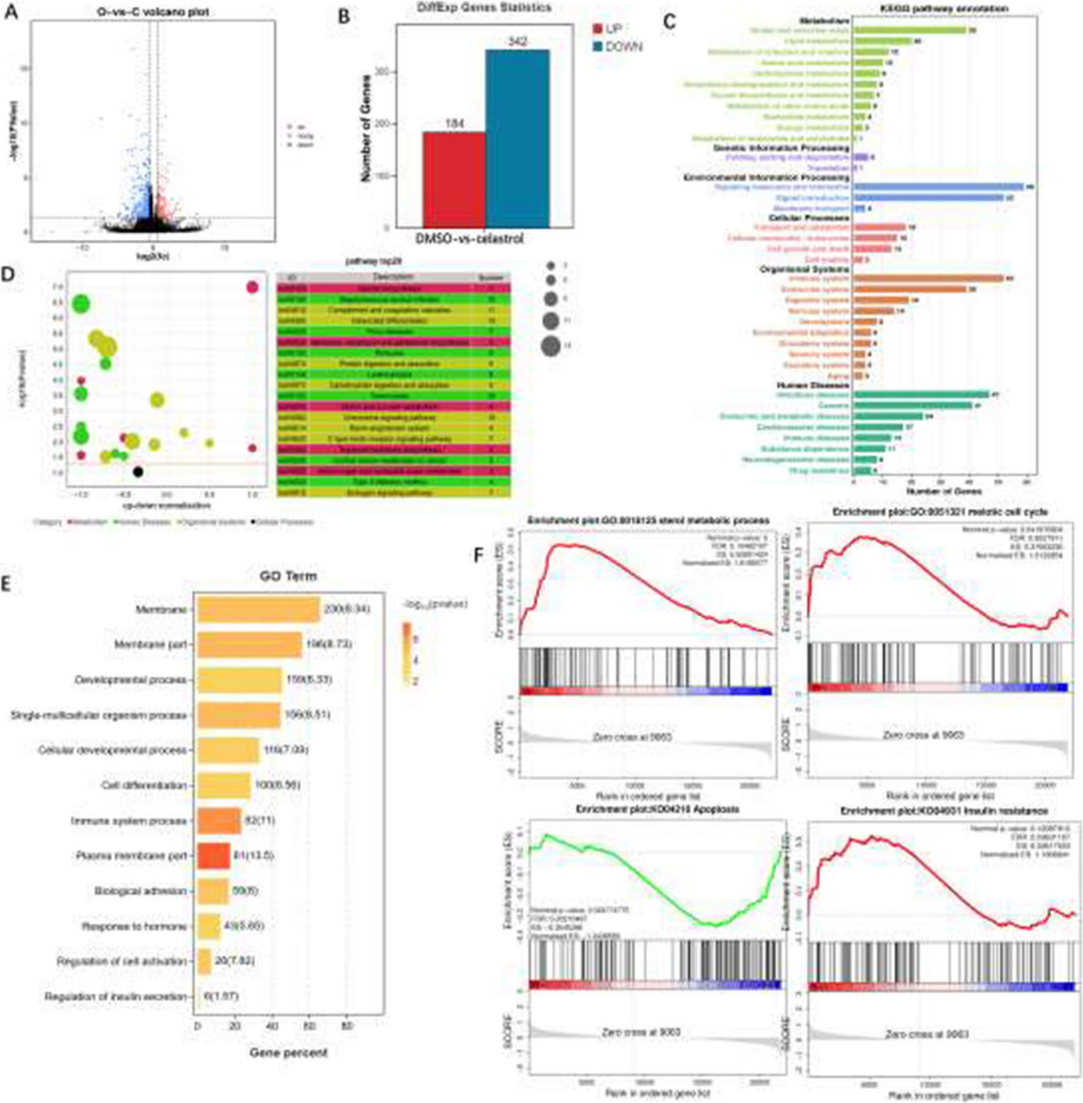


**Fig. 4** Transcriptomic changes induced by celastrol (3 mg/kg) in 12-month-old female mice with 3 weeks of treatment. **A** Volcano plot of DEGs in ovarian tissues from 12-monthold mice treated with celastrol and DMSO (control). **B** Bar chart illustrating the number of upregulated and downregulated DEGs. **C** KEGG functional annotation bar plot of DEGs. **D** KEGG enrichment bubble plot of DEGs. **E** GO pathway enrichment bar chart of DEGs across various pathways. **F**) GSEA analysis of enriched gene sets, with blue and red bars indicating downregulated and upregulated genes, respectively


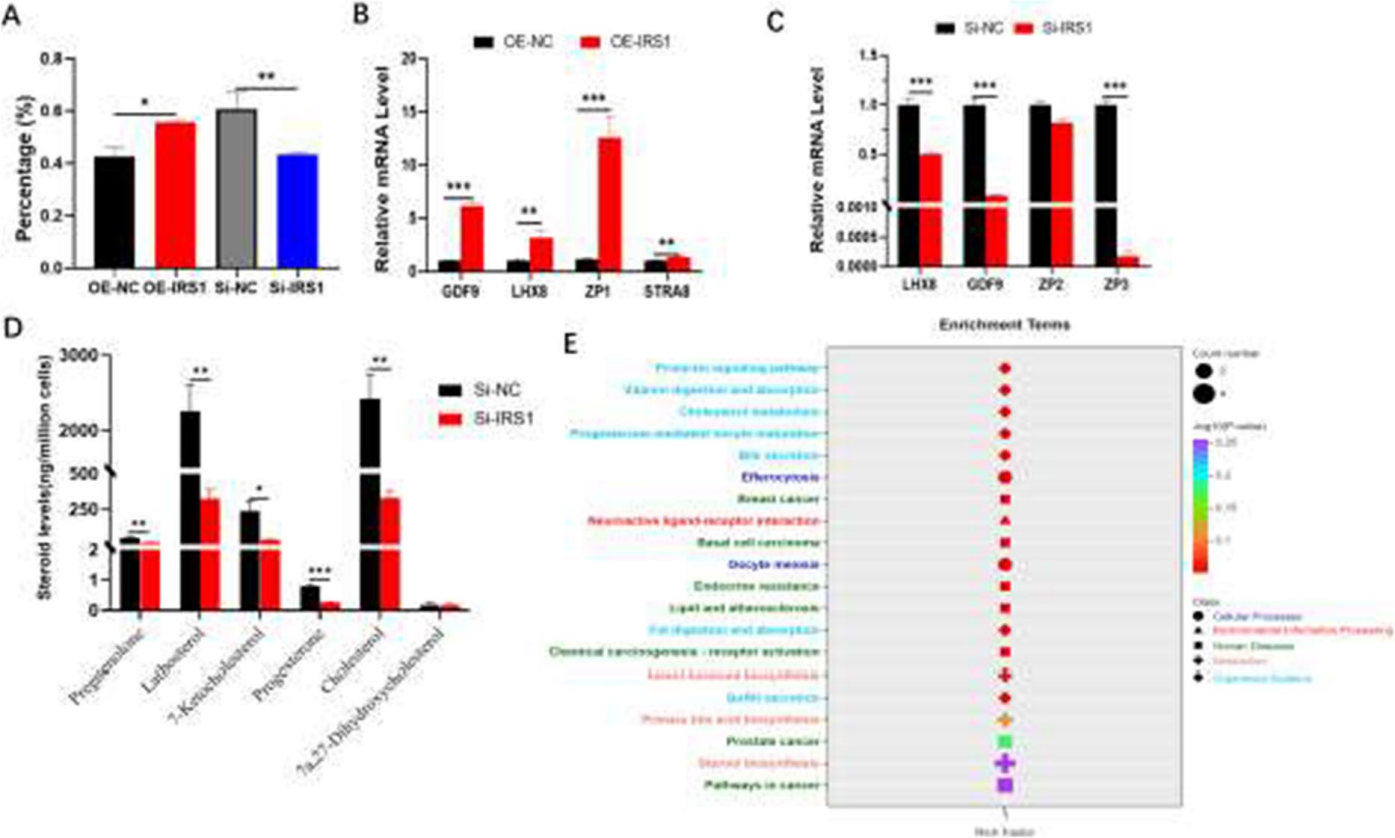


**Fig. 6** Effects of IRS1 on oocyte maturation and steroid hormone changes in porcine GCs. **A** Porcine oocyte maturation rate after in vitro maturation culture. **B**-**C** Changes in mRNA expression levels of oocyte-related genes after small RNA injection. **D** Alterations in steroid hormone levels in GCs following Si-RNA transfection. **E** KEGG pathway analysis of signaling pathways enriched in steroid hormone biosynthesis. GDF9: growth differentiation factor 9; LHX8: LIM homeobox 8; OE-IRS1: Overexpression Insulin Receptor Substrate 1; OE-NC: Overexpression—Negative Control; PCNA: proliferating cell nuclear antigen; Si-IRS1: Small Interfering RNAInsulin Receptor Substrate 1; Si-NC: Small Interfering RNA—Negative Control; STRA8: Stimulated by retinoic acid 8; ZP1: zona pellucida sperm-binding protein 1; ZP2: zona pellucida sperm-binding protein 2; ZP3: zona pellucida sperm-binding protein 3


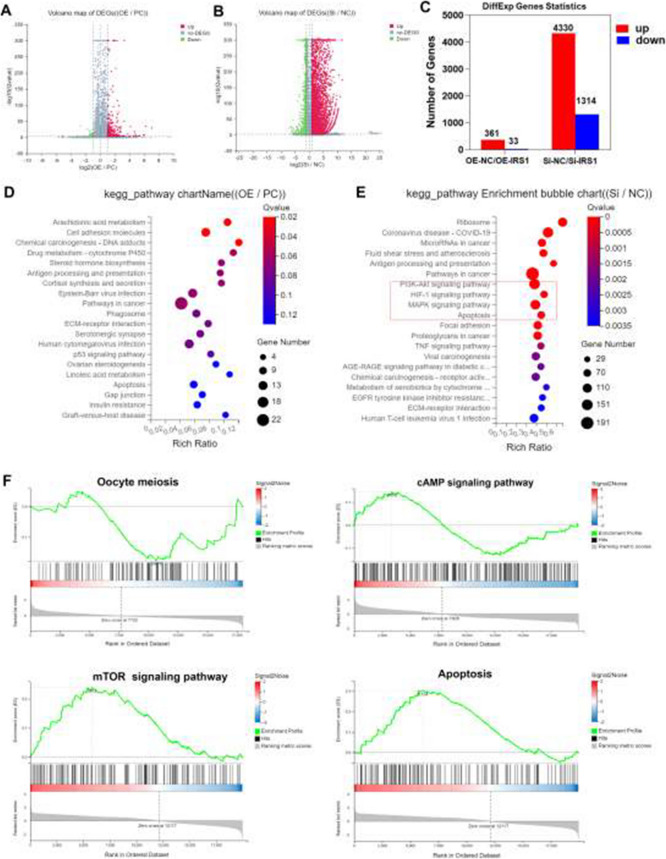


**Fig. 7** IRS1 influences transcriptomic changes in porcine oocytes. **A** Volcano plot of DEGs between the *IRS1*mRNAinjected group and the control group. **B** Volcano plot of DEGs between the si-RNA-injected group and the control group. **C** Bar chart showing the number of DEGs in both treatment groups compared with the control group. **D**-**E** KEGG pathway analysis of enriched signaling pathways associated with DEGs. **F** GSEA enrichment analysis of signaling pathways associated with DEGs


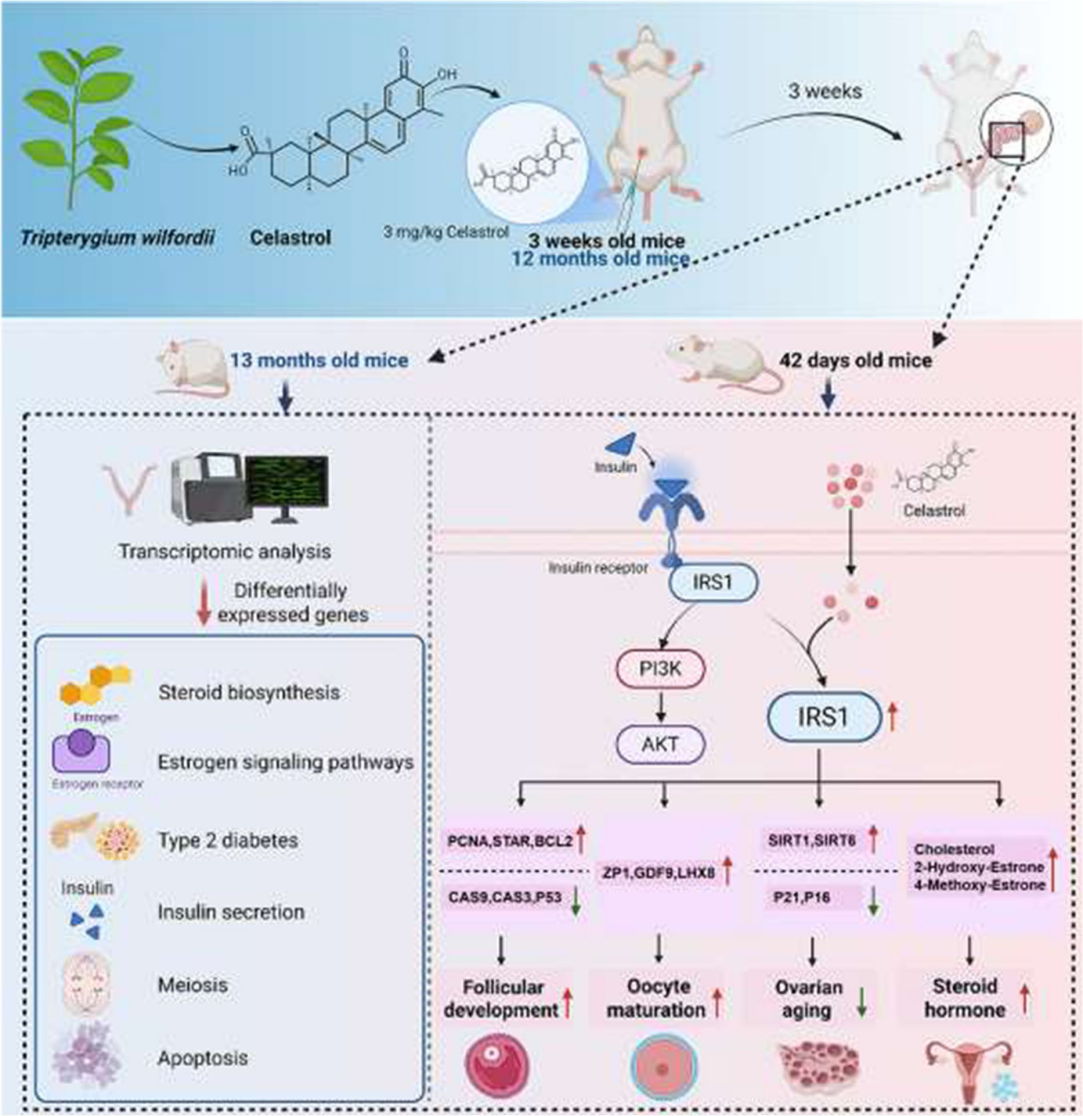


**Fig. 8** Celastrol enhances follicular development and alleviates ovarian aging by modulating IRS1 expression and its related pathways

The original article has been corrected.

