# Supplementary material for: Celastrol modulates IRS1 expression to alleviate ovarian aging and to enhance follicular development
Source: Cell Biol Toxicol. 2025 Sep 2;41(1):129. doi: 10.1007/s10565-025-10079-7 (PMC12405413; doi:10.1007/s10565-025-10079-7)
Supplement: Supplementary file 1 — (PDF 360 KB) [file 10565_2025_10079_MOESM1_ESM.pdf]

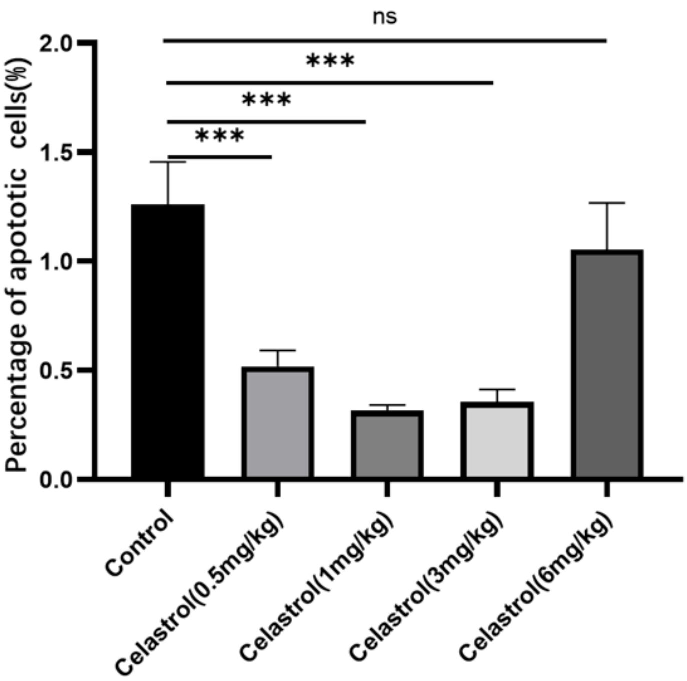

Supplementary Figure 1 TUNEL assay was employed to assess the impact of varying concentrations of celastrol on the apoptosis rate of ovarian GCs of young mice (as shown in Figure 1B). Celastrol was administered to 3-week-old mice via intraperitoneal injection at different concentrations every other day for 3 weeks. After the treatment period, ovarian paraffin sections were prepared, and TUNEL staining was conducted. The fluorescence intensity and percentage of apoptotic cells were quantified using ImageJ. n=3.

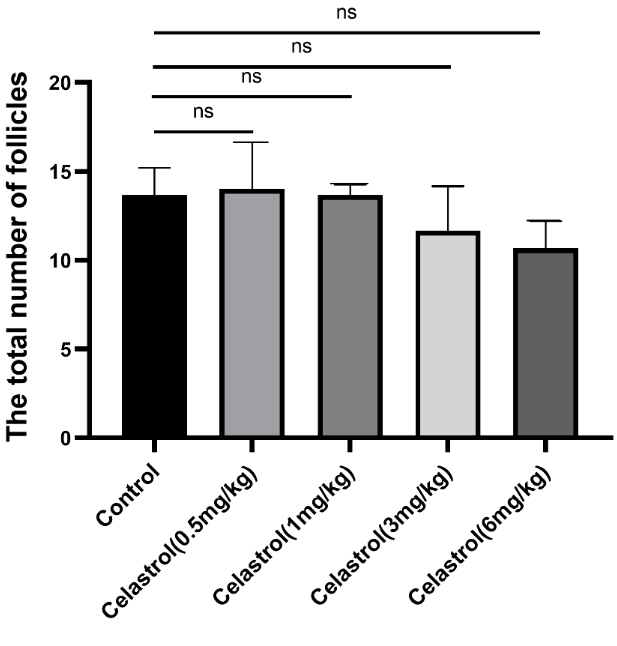

Supplementary Figure 2 Total number of follicles per ovary in young mice with and without celastrol treatment. Celastrol was administered to 3-week-old mice via intraperitoneal injection at various concentrations every other day for 3 weeks. Following the treatment, ovarian paraffin sections were prepared and stained with H&E. The number of follicles was subsequently counted under a microscope. n=3.

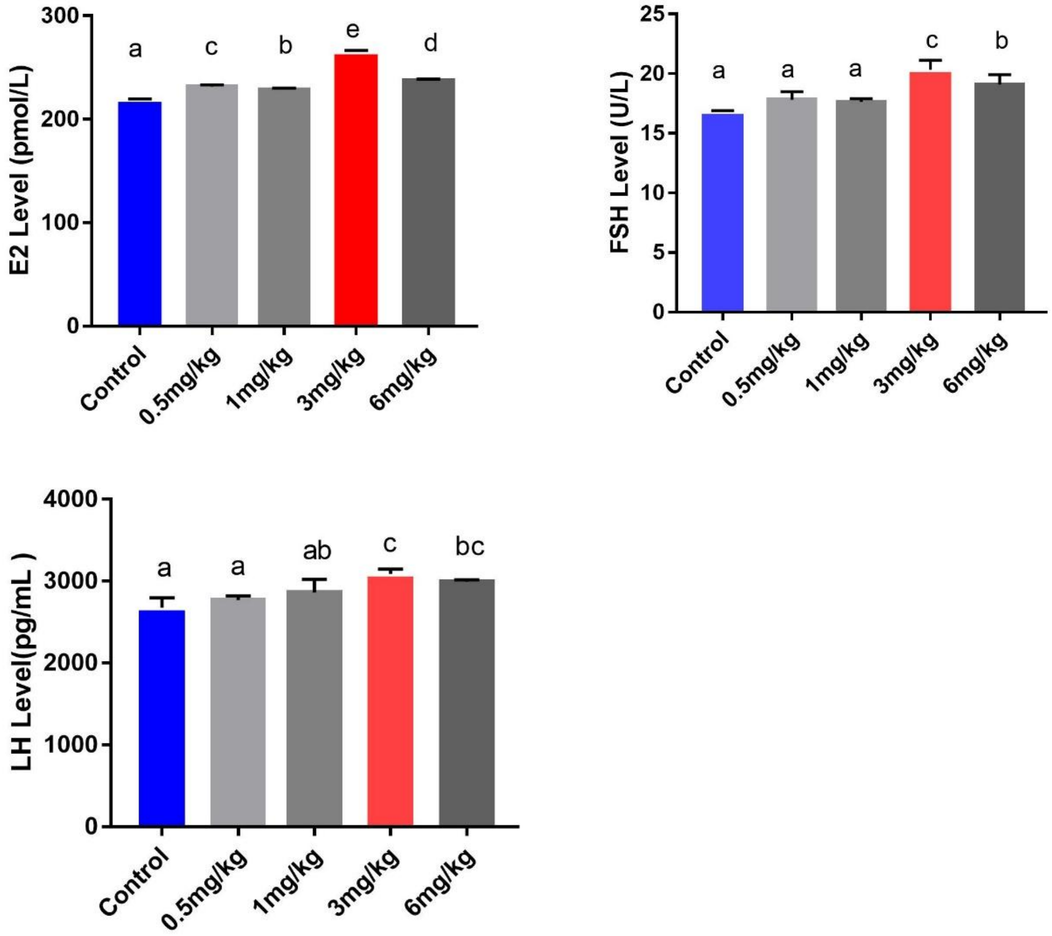

Supplementary Figure 3 Serum levels of E2, FSH, and LH were measured in young mice subjected to celastrol treatment. Celastrol was administered via intraperitoneal injection to 3-week-old mice at varying concentrations every other day for 3 weeks. Following the treatment duration, blood samples were collected, and an ELISA was performed to quantify serum hormone levels. Statistical analysis was conducted using multiple group ANOVA, with a sample size of n=3 for each group.

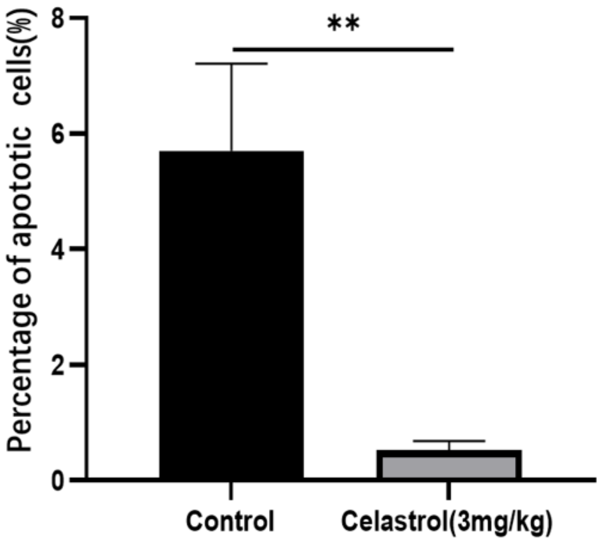

Supplementary Figure 4 TUNEL assay was employed to assess the impact of 3 mg/kg of celastrol on the apoptosis rate of ovarian GCs of middle-aged mice (as shown in Figure 3A). Celastrol was administered to 12-month-old mice via intraperitoneal injection every other day for 3 weeks. After the treatment period, paraffin sections were prepared, and TUNEL staining was conducted. The fluorescence intensity and percentage of apoptotic cells were quantified using ImageJ. n=3.

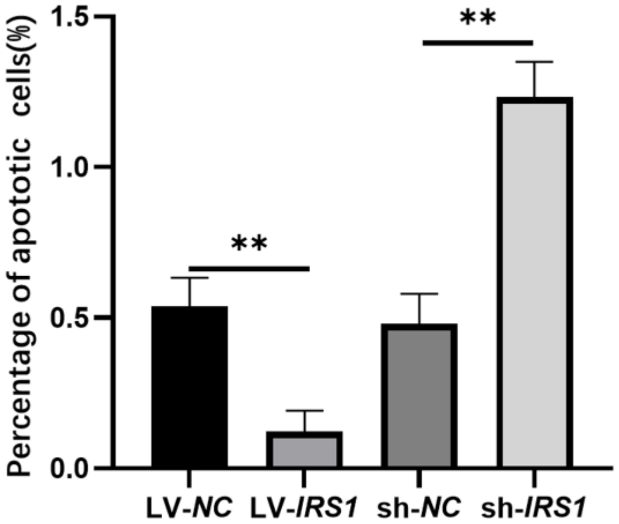

Supplementary Figure 5 TUNEL assay was employed to assess the impact of *IRS1* lentiviral vectors on the apoptosis rate of ovarian GCs in young mice (6 weeks old, comprising 3 weeks of age and 3 weeks of treatment). The study included four groups: the *LV-IRS1* group for IRS1 overexpression, the *LV-NC* group as the negative control, the *sh-IRS1* group for IRS1 knockdown, and the *sh-NC* group as the shRNA negative control. Lentiviral vectors were administered at a dose of 1×10^7 TU every other day via intraperitoneal injection for three consecutive weeks. n=3.

Material and methods

ELISA

Multiple Group Analysis of Variance (ANOVA) was utilized to assess differences among five groups, which included one control group and four treatment groups.

The ELISA assay ranges for the measured hormones are as follows: E2 from 8 pmol/L to 200 pmol/L, FSH from 0.5 U/L to 16 U/L, and LH from 70 pg/ml to 2400 pg/ml. The intra-assay variation for E2 kit is 6.2%, while the inter-assay variation is 8.5%. For LH kit, the intra-assay variation is 5.7% and the inter-assay variation is 7.8%. Lastly, FSH kit exhibits an intra-assay variation of 7.3% and an inter-assay variation of 9.4%.
